# *Plcg2* Deficiency Leads to Sex‐Associated Divergent Effects on Glial Transcriptomes

**DOI:** 10.1002/glia.70252

**Published:** 2026-11-09

**Authors:** Kelly A. Hartigan, Luke C. Dabin, Holly N. Kersey, Nur Jury‐Garfe, Ahmad Daniel Sharify, Byungwook Kim, Bruce T. Lamb, Stephanie J. Bissel, Cristian Lasagna‐Reeves, Jungsu Kim

**Affiliations:** ^1^ Indiana University School of Medicine, Stark Neuroscience Research Institute Indianapolis Indiana USA; ^2^ Indiana University School of Medicine, Medical Neuroscience Graduate Program Indianapolis Indiana USA; ^3^ Department of Medical and Molecular Genetics Indiana University School of Medicine Indianapolis Indiana USA; ^4^ Department of Neurology Baylor College of Medicine Houston Texas USA

**Keywords:** Alzheimer's disease, astrocytes, microglia, neurodegeneration, *Plcg2*, sex differences, single‐cell RNA‐sequencing, transcriptomics

## Abstract

*Plcg2* encodes an intracellular phospholipase C signaling enzyme expressed predominately in microglia in the brain. Genome‐wide association studies implicate *PLCG2* in Alzheimer's disease (AD) risk and loss of *Plcg2* alters amyloid pathology in mouse models. However, the physiological impact of *Plcg2* loss remains unclear. To address this gap, we performed single‐cell RNA sequencing (scRNA‐seq) of wild‐type, heterozygous, and homozygous *Plcg2*‐deficient mouse brains in the absence of amyloid or tau pathology. Our analyses generated a comprehensive, publicly available atlas of glial transcriptomic states across genotypes and sexes. Genotype‐associated changes in microglia and astrocytes were enriched in neurodegenerative disease‐related pathways, as well as oxidative phosphorylation and proteostasis pathways. Genotype‐by‐sex interaction and sex‐stratified analyses revealed divergent regulation of many of these pathways in males and females, while co‐expression and cell–cell communication analyses identified additional sex‐associated differences in glial signaling networks. Overall, these data provide a framework for understanding how variation in PLCG2 function may shape glial biology and modify pathways relevant to neurodegenerative disease in a sex‐associated manner.

## Graphical abstract

- *Plcg2* shapes glial programs linked to neurodegenerative disease–associated pathways in young, pathology‐free brains.
- Microglia and astrocytes exhibit sex‐opposing transcriptomic changes to *Plcg2* deficiency.

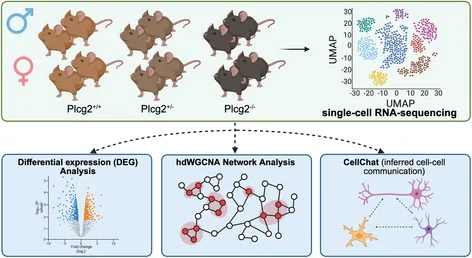

## 1 Background

Neuroinflammation and glial dysfunction are increasingly recognized as central contributors to brain aging and neurodegenerative disease (Heneka et al. 2025; Lee and Landreth 2010; Li et al. 2023). Among glial cells, microglia serve as the resident innate immune cells of the central nervous system and play critical roles in maintaining tissue homeostasis through synaptic remodeling, debris clearance, and regulation of inflammatory signaling (Borst et al. 2021; Bohlen et al. 2019). Disruption of these functions has been implicated across a broad range of neurological conditions, including Alzheimer's disease (AD), Parkinson's disease, and other neurodegenerative disorders (Heneka et al. 2025; Tansey and Romero‐Ramos 2019).

One immune‐associated gene that has gained increasing attention in these fields is phospholipase C gamma 2 (*PLCG2*), a key regulator of intracellular immune signaling. PLCG2 encodes an enzyme that hydrolyzes phosphatidylinositol 4,5‐bisphosphate (PIP2) into second messengers involved in calcium mobilization and downstream signal transduction (Jackson et al. 2021; Magno et al. 2021). In peripheral immune cells, PLCG2 regulates essential functions such as antibody production, inflammation, and pathogen clearance. Additionally, germline mutations in *PLCG2* can cause immune dysregulation syndromes (Jackson et al. 2021; Baysac et al. 2024). Within the brain, *Plcg2* expression is highly enriched in microglia of the parenchyma, positioning it as a key immune signaling molecule in the CNS.

Multiple variants in *PLCG2* have been linked with altered risk for late‐onset AD (Turner et al. 2026; Kleineidam et al. 2020; Claes et al. 2022; Diks et al. 2023; Magno et al. 2019; Takalo et al. 2020; Tsai et al. 2023; Li et al. 2022; Oblak et al. 2022), including two commonly studied rare coding variants. The mildly hypermorphic *PLCG2* ^*P522R*^ variant is associated with longer lifespan and reduced AD risk (Kleineidam et al. 2020; Sims et al. 2017). It is also associated with enhanced Aβ clearance (Kleineidam et al. 2020; Claes et al. 2022; Diks et al. 2023; Magno et al. 2019; Takalo et al. 2020; Tsai et al. 2023). Conversely, the *PLCG2* ^*M28L*^ loss‐of‐function variant has been reported to show a nominal association with increased AD risk and exacerbates Aβ‐related phenotypes in experimental models (Tsai et al. 2023; Li et al. 2022; Oblak et al. 2022). Similarly, a recent study reported that *Plcg2* deletion increases plaque size and decreases microgliosis in a mouse model of amyloid pathology (Messenger et al. 2025). Current research often positions PLCG2 downstream of TREM2 signaling, a pathway strongly implicated in AD risk (Messenger et al. 2025; Andreone et al. 2020; Obst et al. 2021; Bedford et al. 2025). These prior studies underscore the importance of *Plcg2* in disease‐associated processes. However, these observed phenotypes may arise, in part, from disruptions of its physiological functions. Therefore, a clear understanding of the function of *Plcg2* under homeostatic conditions is essential for interpreting its contributions to pathology. Nevertheless, the physiological functions of *Plcg2* remain incompletely understood.

Bulk‐omic studies indicate that *Plcg2* loss alters immune gene expression, suggesting a key role in microglial immune response (Obst et al. 2021; Bedford et al. 2025), with prominent changes more readily observed in females (Tsai et al. 2022; Staley et al. 2024). Studies using induced pluripotent stem cell‐derived microglia‐like cells recapitulate these findings (Andreone et al. 2020). In these studies, female microglia lacking *PLCG2* show modified cytokine expression in response to immune stimulation (Bedford et al. 2025). However, it remains unclear whether *Plcg2* affects baseline microglial gene expression states, whether these effects vary by sex, and whether *Plcg2* is associated with changes in other glial populations.

This study addresses these gaps by applying single‐cell RNA sequencing (scRNA‐seq) to brains of male and female mice from wild‐type (WT), *Plcg2* heterozygous knockout (HET), and *Plcg2* homozygous knockout (KO) backgrounds. Utilizing this single‐cell resolution approach, we determined how *Plcg2* perturbation is associated with changes in microglial heterogeneity in both male and female glia under physiological conditions. By integrating differential gene expression, pathway, co‐expression, and cell–cell communication analyses, we identify coordinated glial expression programs associated with *Plcg2* deficiency, including programs that overlap with pathways relevant to neurodegenerative disease. Together, these findings establish a baseline transcriptomic framework for understanding the consequences of *Plcg2* perturbation in the mouse brain.

## 2 Methods

### 2.1 Animals

All experiments were approved by the Institutional Animal Care and Use Committee at Indiana University. Mice were housed and maintained in a 12‐h/12‐h light/dark cycle with food (Purina Lab Diet, 5K52) and water ad libitum. B6.Cg‐Plcg2em2Adiuj/J (*Plcg2* KO; The Jackson Laboratory, 029910) were used to breed heterozygous and homozygous knockout mice as well as wild‐type littermates. Genotyping protocol was performed as described by The Jackson Laboratory.

### 2.2 Single‐Cell RNA Library Preparation

Cell isolation was performed as previously described (Dabin et al. 2024). Briefly, 6‐week‐old mice were anesthetized and transcardially perfused with ice‐cold PBS. Brains were dissected, and the right hemisphere (excluding the cerebellum and olfactory bulb) was dissociated in StemPro Accutase (Fisher Scientific), followed by gentle mechanical trituration and sequential filtration through 70 and 40 μm strainers. Cell suspensions were centrifuged, and myelin debris was removed using anti‐myelin magnetic beads and LS columns (Miltenyi). The resulting myelin‐depleted single‐cell suspensions were washed, resuspended in PBS with 0.04% BSA, and viability was assessed by trypan blue staining. Single‐cell suspensions were then processed using the 10x Genomics Chromium Next GEM v3.1 Single Cell 3′ dual index kit for barcoding and library preparation. The cDNA and library quality were assessed using the Agilent 2100 Bioanalyzer and a High Sensitivity DNA kit (Agilent Technologies, Santa Clara, CA), and the final library concentration was determined using a Qubit Fluorometer and the dsDNA HS assay kit (Thermo Fisher Scientific, Waltham, MA). A total of 14 mice (4 WT, 5 HET, 5 KO; 2 female, 2–3 male per genotype) were used for single‐cell RNA sequencing (Figure S1A). Prepared libraries were sequenced on an Illumina NovaSeq 6000 platform, targeting 50,000 reads per cell per sample.

### 2.3 Single‐Cell RNA‐Sequencing Data Analysis

Processing of the sequencing data was performed with the cellranger pipeline (8.0.1, 10× Genomics). The filtered feature‐cell barcode matrices generated by CellRanger were loaded into SoupX (Young and Behjati 2020) (1.6.0) in RStudio (2025.09.0) running R (4.4.0) and cleaned using default settings. Cleaned data were then loaded into Seurat (Hao et al. 2024) (5.0.3). During quality control, cells with > 10% mitochondrial reads or unique reads below 200 were excluded from analysis. Data were normalized using SCTransform (0.4.1), and UMAP dimensional reduction and clustering were performed using the first 50 principal components and a resolution of 0.6. Cluster annotation was performed using the Seurat FindAllMarkers function and were manually annotated based on marker expression in conjunction with EnrichR (Kuleshov et al. 2016). Differences in proportion of cells per cluster analysis was performed using the R package Speckle (Phipson et al. 2022) (1.4.0). The variancePartition (Hoffman and Schadt 2016) R package was used to estimate the fraction of gene expression variance explained by sex, genotype, and sex‐by‐genotype interaction effects across samples with sequencing run (included as the technical‐batch covariate) removed as a technical variable. Prior to variance‐partitioning analysis, single‐cell counts were aggregated into one pseudobulk expression profile per mouse. Pseudobulked differential expression analysis and genotype‐by‐sex interaction effect was performed as follows: within each cell type, raw counts were summed across cells from the same animal to generate one pseudobulk expression profile per mouse. Prior to differential expression analysis, genes with low or inconsistent expression across the pseudo‐bulked microglia samples were removed. Counts per million (CPM) were calculated for each gene across all pseudobulk samples using the cpm() function from the edgeR (Robinson et al. 2010) package for the purpose of filtering low quantity transcripts. Genes were tested only if it had a CPM greater than 5 in at least 75% of samples. Genes were then further filtered to include only protein‐coding genes. After filtering, 9322 genes were retained for microglia and 9924 for astrocytes. Differential expression across genotypes was analyzed using DESeq2 (Love et al. 2014) with the ~ sex + technical batch + genotype, with WT designated as the reference levels. The genotype‐by‐sex interaction across genotypes was analyzed as ~sex:genotype + sex + genotype + technical batch, with WT and female designated as the reference levels. The genotype‐by‐sex interaction coefficients therefore represented the difference between the male and female genotype effects. For example, the KO‐by‐sex coefficient was defined as (KO male − WT male) − (KO female − WT female). Wald tests were used to evaluate the KO‐by‐sex and HET‐by‐sex interaction coefficients separately. *p* values were adjusted across all genes tested within each cell type and contrast using the Benjamini–Hochberg procedure. Genes with an adjusted *p* value below 0.05 were considered statistically significant. Cell‐level differential gene expression analysis was performed using a Model‐based Analysis of Single‐cell Transcriptomics (Finak et al. 2015) (MAST; 1.30.0) function, with regression of percent ribosomal RNA, percent mitochondrial RNA, and sequencing depth. Because this cell‐level MAST analysis did not explicitly model mouse identity, it was treated as an exploratory analysis and was not used for primary sample‐level inference. CellChat (Jin et al. 2025) (2.1.2) was used to infer inter‐cluster signaling using default package settings. High‐dimensional weighted gene co‐expression network analysis (hdWGCNA) was performed using hdWGCNA (Morabito et al. 2023) (0.3.03). For hdWGCNA analysis, metacells were constructed by aggregating transcriptomically similar cells originating from the same biological sample and annotated cell type, thereby ensuring that co‐expression networks were computed within cell‐type‐specific expression space. Gene Set Enrichment Analysis (GSEA) and Over‐representation analysis (ORA) was run using ClusterProfiler (Wu et al. 2021) (4.12.0) using Kyoto Encyclopedia of Genes and Genomes (KEGG) and MSigDB Hallmark gene set collection (Liberzon et al. 2015). For rank‐based GSEA, genes were ranked by the signed Wald statistic from the corresponding DESeq2 contrast. To reduce bias from constitutive cell‐type specific expression programs, ORA was performed using genes detected within the corresponding microglial or astrocyte populations as the background reference.

Raw data have been deposited in the NIH GEO database, scRNA‐seq data (GSE313890).

## 3 Results

### 3.1 *Plcg2* Is Co‐Expressed With Multiple AD Risk Genes

Prior studies of *Plcg2* in the brain have primarily focused on AD pathology or AD risk associated alleles (Turner et al. 2026; Kleineidam et al. 2020; Claes et al. 2022; Diks et al. 2023; Magno et al. 2019; Takalo et al. 2020; Tsai et al. 2023; Li et al. 2022; Oblak et al. 2022). However, understanding *Plcg2*'s normal physiological functions is essential for placing its disease‐associated roles in a proper mechanistic context. To investigate the role of *Plcg2* in a physiological context, we generated single‐cell RNA‐seq (scRNA‐seq) datasets from male and female *Plcg2* ^*+/+*^ (WT), *Plcg2* ^*+/−*^ (HET), and *Plcg2* ^*−/−*^ (KO) mice (2–3 per sex per genotype; total *N* = 14). After stringent quality control measures (see Methods), we retained 62,303 cells in our dataset for downstream analysis. We identified 27 transcriptomically distinct clusters through dimensionality reduction and clustering using PCA and UMAP (Figure 1A). We annotated clusters by canonical marker expression as microglia (M), peripheral immune cells (PI), astrocytes (A), ependymal cells (Ep), endothelial cells (En), red blood cells (RBC), and few remaining neurons (N; Figure 1B, Figure S1A, Tables S1 and S2). Analysis of *Plcg2* expression across WT cell populations demonstrated that expression was overwhelmingly enriched in microglial and peripheral immune populations, with minimal to absent expression in other cell types (Figure S1B). Microglia represented the largest cell population and segregated into 11 transcriptomically distinct clusters (0_M, 1_M, 2_M, 3_M, 4_M, 5_M, 6_M, 8_M, 9_M, 13_M, and 17_M). Clusters were named according to the number of cells per cluster, with 0_M being the largest and 17_M being the smallest. Genotypes showed no significant differences in the proportions of major cell types (Figure 1C and Table S3). Similarly, the relative abundance of most microglial clusters remained stable across genotypes. However, female HET mice showed a significant reduction in cluster 5_M compared with WT samples (adjusted *p* = 0.034, Figure S1 and Table S4).

**FIGURE 1:**
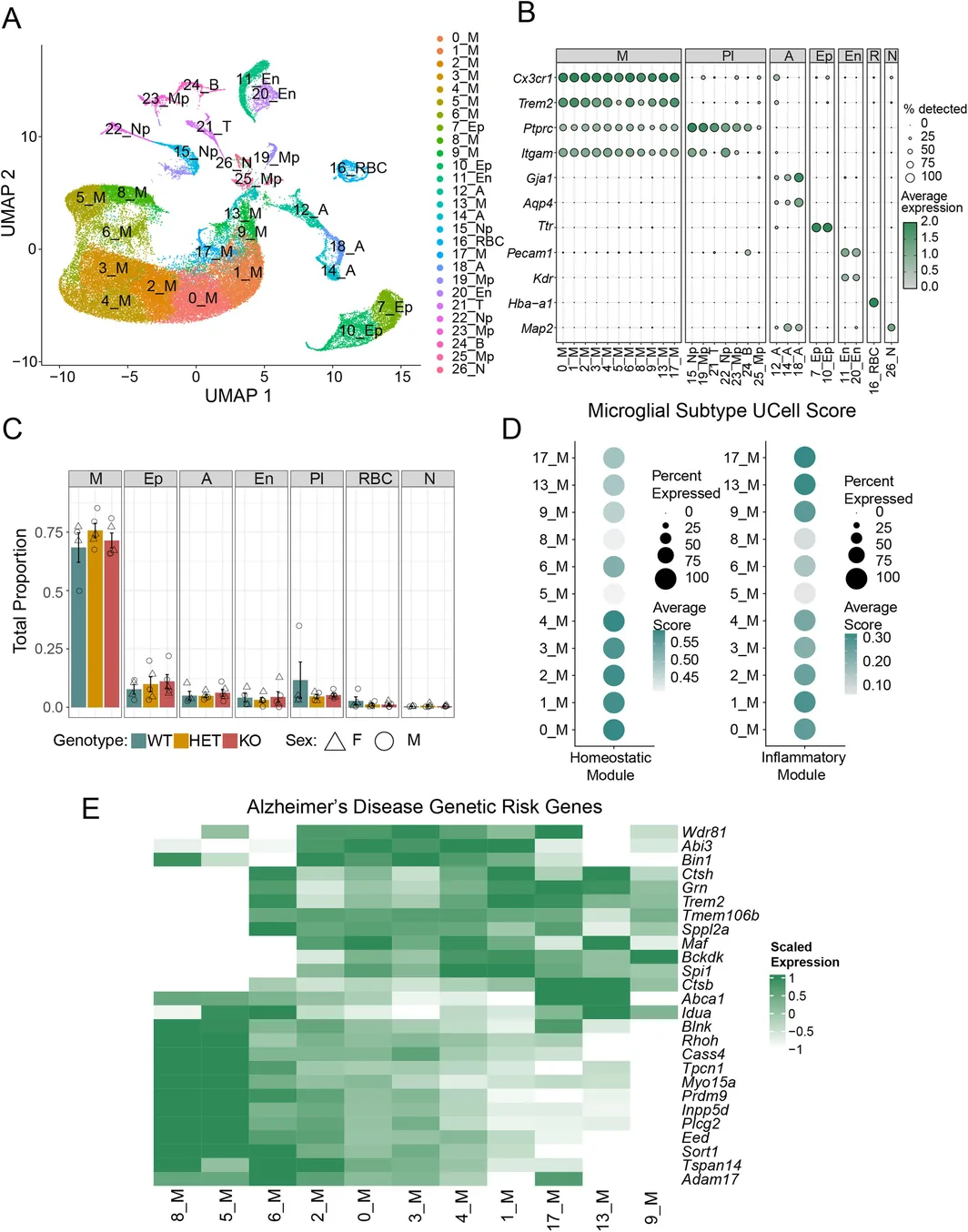
*Plcg2* is co‐expressed with Alzheimer's disease risk genes in young, healthy mice. (A) Annotated Uniform Manifold Approximation and Projection (UMAP) plot of 62,303 cells after quality control including microglia (M), astrocytes (A), ependymal cells (Ep), endothelial cells (En), red blood cells (RBC), neutrophils (Np), macrophages (Mp), T‐Cells (T), B‐Cells (B), and neurons (N). (B) Dot plot displaying expression of canonical marker genes for each major cell type cluster, including microglia (*Cx3cr1*, *Trem2*), peripheral immune cells (*Ptprc*, *Itgam*), astrocytes (*Aqp4*, *Gja1*), ependymal cells (*Ttr*), endothelial cells (*Pecam1*, *Kdr*), red blood cells (*Hba‐a1*), and neurons (*Map2*). Dot size indicates percent of cells expressing each marker, color represents average expression per cell. (C) The mean proportion of each cell type captured relative to the total population based on genotype, WT (*Plcg2+/+*), HET (*Plcg2+/−*) and KO (*Plcg2−/−*). Error bars represent standard error of the mean (SEM). Individual samples are overlaid as points, with triangles denoting females and circles denoting males. (D) Microglial subcluster UCell module scores for homeostatic‐ and inflammatory‐associated gene signatures. Dot size indicates the percent of cells expressing each module, color represents average module score per cell. (E) Heatmap of scaled *z*‐score expression of key Alzheimer's disease genetic risk genes across microglial clusters.

Given the well‐established heterogeneity of microglial states (Fumagalli et al. 2025; Depp et al. 2025; Wang 2021), we next sought to annotate the transcriptional states of all microglial clusters using module scoring with previously defined microglial gene signatures (Fumagalli et al. 2025). We found that clusters 0_M, 1_M, 2_M, 3_M, 4_M, and 6_M exhibited high enrichment for homeostatic signatures, whereas clusters 9_M, 13_M, and 17_M were primarily enriched for inflammatory signatures (Figure 1D and Figure S2A,B). Clusters 5_M and 8_M did not show strong enrichment for either homeostatic or inflammatory signatures. Because *Plcg2* has been implicated in AD risk, we next evaluated how its expression overlaps with other microglial AD risk genes. We compiled AD risk genes from a recent large‐scale GWAS (Bellenguez et al. 2022) and evaluated their expression across the major cell types in our dataset. Genes were classified as microglia‐enriched when their mean log‐normalized expression in microglia was greater than the mean expression observed in each of the other identified cell types, resulting in 26 risk‐associated genes. We then examined the expression of these genes across the identified microglial clusters. For each gene and cluster, we quantified both the percentage of cells with detectable expression (> 0) and the mean unscaled log‐normalized expression (Table S5). These analyses demonstrated substantial heterogeneity in the expression of AD risk‐associated genes across microglial clusters. Scaled expression profiling revealed that *Plcg2* expression was highest in clusters 5_M and 8_M. Interestingly, these clusters also showed co‐expression with several other AD risk genes, including *Inpp5d* and *Blnk* (Figure 1E). To further elucidate the states of these two microglial clusters, we ran pathway enrichment for marker genes of clusters 5_M and 8_M. This analysis revealed a unique RHO GTPase signature in both clusters 5_M and 8_M (Figure S2C,D; Tables S6 and S7). Collectively, these analyses identify microglial populations in which *Plcg2* is co‐expressed with several AD risk‐associated genes, including genes with both established and candidate functional relationships to *PLCG2*.

### 3.2 Genotype‐Dependent Transcriptomic Changes in Microglia

Differential expression analysis of *Plcg2* KO versus WT microglial identified 100 genes meeting the prespecified BH‐adjusted significance threshold (Figure 2A and Table S8), after adjusting for sex and technical variables. Downregulated genes included activation‐associated microglial transcripts (*Cd83*, *Plaur*, *Ccl4*, *Cst7*) alongside the Rac‐GEF adaptor *Ipcef1*, consistent with attenuated immunoreceptor‐proximal and cytoskeletal signaling, while upregulated genes were dominated by interferon‐stimulated genes (*Gbp7*, *Rnasel*, *Trim30b*, *Sp100*, *Rtp4*) along with the stress‐ and transcription‐associated regulators *Nupr1* and *Jdp2*. To evaluate coordinated pathway‐level genotype effects, genes were ranked by the signed Wald statistic from the sex‐adjusted KO‐versus‐WT pseudobulk model and analyzed by gene set enrichment analysis (GSEA). GSEA using KEGG gene sets revealed enrichment of pathways related to ribosome function, oxidative phosphorylation, and several canonical neurodegenerative disease signatures (Huntington's, Parkinson's, Alzheimer's, ALS, prion disease), all with negative Normalized Enrichment Score (NES), indicating coordinate downregulation of these programs in the *Plcg2* KO (Figure 2B and Table S9). MSigDB Hallmark GSEA reveals downregulation of programs associated with cholesterol homeostasis and reactive oxygen species pathways but an increase in interferon gamma response programs (Figure 2C and Table S9). By comparison, the HET versus WT contrast yielded a much more modest differential gene expression signature (Figure 2D and Table S8), with only E3 ubiquitin‐protein ligase *Rnf150* reaching multiple comparison corrected significance. This is consistent with a potential gene‐dosage effect in which heterozygous loss of *Plcg2* may produce an intermediate, attenuated phenotype relative to full knockout. Despite this attenuated response, KEGG GSEA showed negative enrichment of oxidative phosphorylation and similar neurodegenerative disease pathways (Figure 2E and Table S10). Hallmark GSEA further revealed negative enrichment of MYC/E2F targets, DNA repair, mTORC1 signaling, glycolysis, and fatty acid metabolism (Figure 2F and Table S10). Together, these findings suggest that heterozygous loss of *Plcg2* produces a weaker but partially concordant pathway‐level phenotype relative to complete knockout.

**FIGURE 2:**
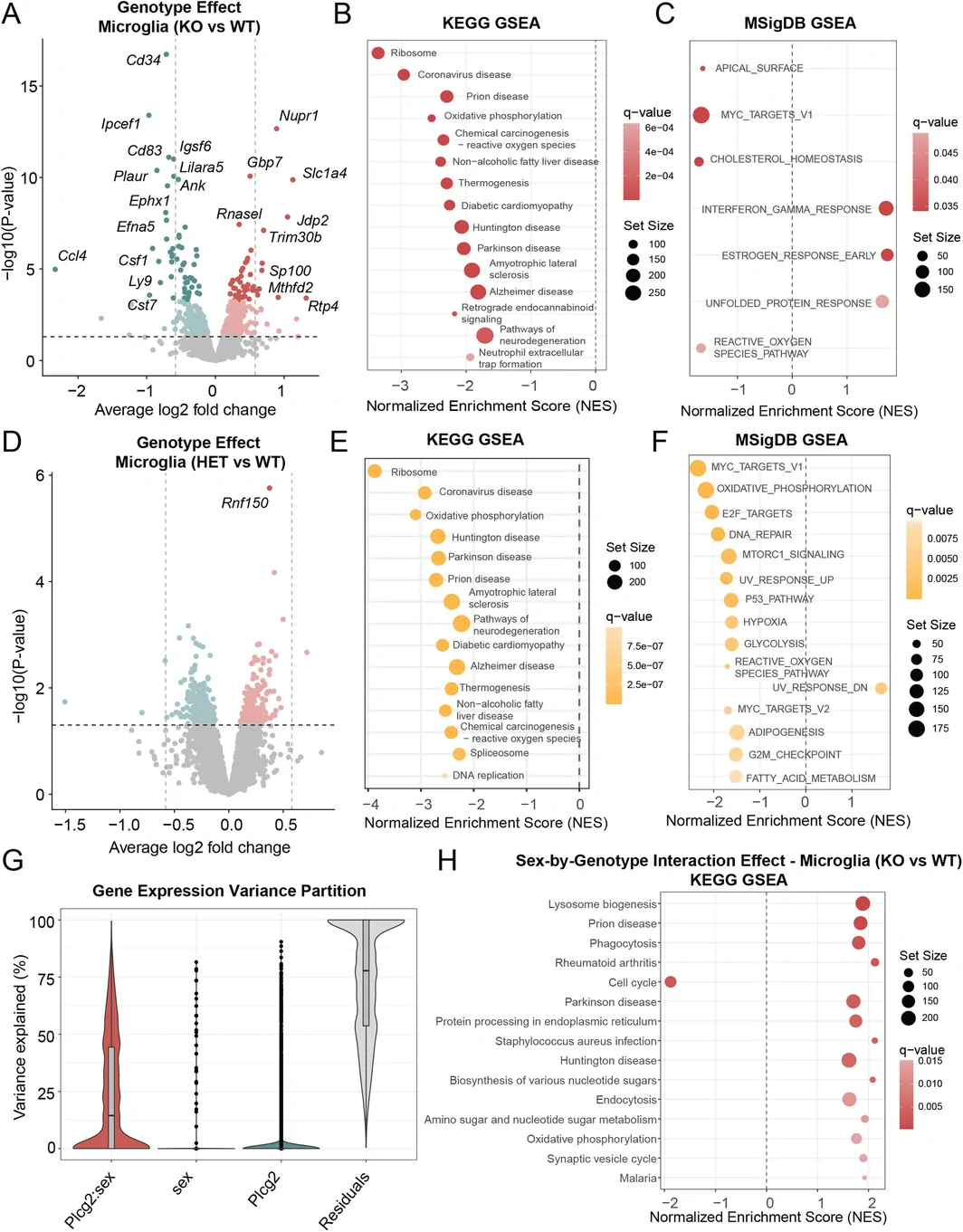
*Plcg2* genotype effects and genotype‐by‐sex interaction analyses in microglia. (A, D) Volcano plots of pseudobulk differential‐expression results in microglia comparing *Plcg2* KO versus WT (A) and HET versus WT (D). Genes meeting the nominal threshold (*p* value < 0.05, |log2FC| > 0.58) are highlighted, with select genes labeled; genes additionally reaching a Benjamini‐Hochberg‐adjusted *p* value < 0.05 are shown in darker color. Red indicates genes upregulated in KO and HET; blue indicates genes downregulated in KO and HET. (B, C) Gene set enrichment analyses (GSEA) of genes ranked by the signed Wald statistic from the KO versus WT comparison. (B) KEGG and (C) MSigDB Hallmark gene sets. (E, F) GSEA of genes ranked by the signed Wald statistic from the HET versus WT comparison for (E) KEGG and (F) MSigDB Hallmark gene sets. For (B, C, E, F), the *x*‐axis represents the normalized enrichment score (NES); dot size indicates gene set size, and color indicates *q*‐value. (G) Violin plots show the distribution of transcriptome‐wide variance explained (%) for sex, *Plcg2* genotype, a sex‐by‐genotype interaction term (*Plcg2*:sex), and residual unexplained variance, with embedded boxplots indicating median and interquartile range. (H) KEGG GSEA of genes ranked by Wald statistic for the KO‐by‐sex interaction in pseudobulk microglia; dot size indicates gene set size and color indicates *q*‐value.

To quantify the relative contribution of sex, genotype, and their interaction to gene expression variability, we performed gene‐wise variance decomposition across all microglia using the variancePartition (Hoffman and Schadt 2016) R package. For many genes, the sex‐by‐genotype term accounted for a substantial fraction of expression variance relative to the main effects of sex or genotype (Figure 2G). This pattern motivated formal testing of genotype‐by‐sex interactions using mouse‐level pseudobulk models. However, formal mouse‐level pseudobulk analysis did not identify individual genes with a significant genotype‐by‐sex interaction after multiple‐testing correction for either the KO versus WT or HET versus WT comparisons (Benjamini–Hochberg‐adjusted *p* > 0.05; Table S11). Detecting interaction effects at the gene level requires substantially greater statistical power than detecting main genotype effects (Leon and Heo 2009; Brookes et al. 2004). Due to our limited numbers of biological replicates within each sex‐by‐genotype group, we are likely underpowered to detect these differences at the sample level. We therefore reasoned that coordinated sex‐dependent effects might be more readily detectable at the pathway level than for individual genes and performed GSEA of the genotype‐by‐sex interaction. Interaction GSEA identified coordinated sex‐associated differences in the transcriptomic response to *Plcg2* loss across several biologically relevant pathways, despite the absence of individual genes surviving multiple‐testing correction. Following complete *Plcg2* loss, KO‐associated effects on phagolysosomal pathways, including lysosome biogenesis, phagocytosis, and endocytosis, were greater in males than females, alongside enrichment of neurodegeneration‐associated pathways such as Parkinson and prion disease (Figure 2H and Table S12) as well as oxidative phosphorylation and interferon response (Figure S3A and Table S12). In contrast, HET microglia showed predominantly negative interaction enrichment for cell‐cycle, metabolic, and neurodegeneration‐associated pathways, including oxidative phosphorylation and Alzheimer, Parkinson, Huntington, ALS, and prion disease pathways (Figure S3B,C and Table S13). These findings provide evidence for coordinated pathway‐level patterns associated with the genotype‐by‐sex interaction statistic despite the absence of FDR‐significant individual‐gene interactions.

### 3.3 *Plcg2* Loss Induces Distinct Transcriptomic Changes in Female and Male Microglia at the Cell Level

Given the variance‐partitioning results and pathway‐level enrichment associated with the formal interaction statistic, we next examined genotype‐associated expression patterns separately in male and female microglia. While interaction testing formally assesses whether genotype effects differ by sex, it can be underpowered to detect biologically meaningful differences at the individual‐gene level in the context of a small, preclinical cohort. Sex‐stratified differential expression and pathway analyses provide a complementary approach, allowing us to define the transcriptomic consequences of *Plcg2* perturbation within each sex and identify responses that may not be apparent in the combined analysis. In this way, sex‐stratified analyses illuminate the gene‐expression patterns underlying the pathway‐level interaction signals, while remaining distinct from a formal test of interaction.

In our female cohort, sex‐stratified pseudobulk analysis revealed a more pronounced genotype‐associated effect of *Plcg2* loss in KO than HET microglia (Figure S4A,C and Table S14). In HET females, KEGG GSEA identified both positively and negatively enriched pathways, including negative enrichment of prion disease, proteasome, and antigen processing and presentation pathways (Figure S4B and Table S15), while MSigDB Hallmark GSEA showed no significant results. In contrast, KO females showed strong negative enrichment of ribosomal and oxidative phosphorylation pathways and multiple neurodegenerative disease signatures (Figure S4D and Table S16). MSigDB Hallmark GSEA similarly identified reduced oxidative phosphorylation and inflammatory signaling pathways, including TNFα/NF‐κB and IL6/JAK/STAT3 pathways (Figure S4E and Table S16). Together, these findings indicate a stronger and more coordinated pathway‐level response to complete *Plcg2* loss in female microglia.

While pseudobulk analysis provides the appropriate framework for mouse‐level statistical inference, aggregation across cells can obscure the breadth and heterogeneity of transcriptomic changes within the microglial population. We therefore complemented our pseudobulk analyses with exploratory cell‐level differential expression analyses to more comprehensively determine the gene expression changes in the context of partial versus complete *Plcg2* loss in females. Because this cell‐level analysis does not account for biological replication at the mouse level, these findings should be considered exploratory and interpreted alongside the sample‐level pseudobulk analyses. Comparing HET and KO microglia with sex‐matched WT controls revealed widespread transcriptomic changes in both genotypes (Figure 3A–B and Table S17). Comparison of HET and KO differential expression profiles revealed both shared and genotype‐specific transcriptomic changes. A total of 2598 DEGs were shared between HET and KO microglia, while 1223 genes were uniquely dysregulated in HET animals and 711 genes were unique to KO animals (Figure 3C). To determine whether these shared DEGs were concordantly or discordantly regulated between genotypes, we generated an UpSet plot in which each intersection is split by direction of change relative to WT (Figure 3D and Table S18). This analysis showed that the vast majority of overlapping DEGs (2542/2598, 97.8%) were concordantly regulated with only 56 genes (2.2%) showing discordant directionality between genotypes. Shared DEGs also demonstrated highly concordant effect sizes between HET and KO conditions when compared to WT (Pearson *r* = 0.929, *R* ^2^ = 0.863). Furthermore, linear regression indicated that HET effect sizes closely approximated those observed in KO animals (slope = 0.954, 95% CI: 0.939–0.968; Figure S5A). We next assessed monotonic expression changes by identifying genes exhibiting concordant directional changes in both HET and KO animals, with greater effect magnitude in KO relative to HET comparisons. Using these criteria, we identified 1247 monotonic genes in female microglia (Figure S5A and Table S19). Together, these findings indicate that the cell‐level expression changes associated with heterozygous *Plcg2* loss substantially overlap those observed following complete deletion in females. However, the presence of genotype‐specific DEG subsets suggests that complete loss of *Plcg2* additionally induces distinct transcriptomic programs not fully recapitulated in the heterozygous state. To more directly compare gene expression changes between HET and KO animals, we performed differential expression analysis between female KO and HET microglia (Figure S5B and Table S17). Consistent with the overlap observed in shared DEGs, direct genotype comparison identified comparatively modest transcriptomic changes between HET and KO animals.

**FIGURE 3:**
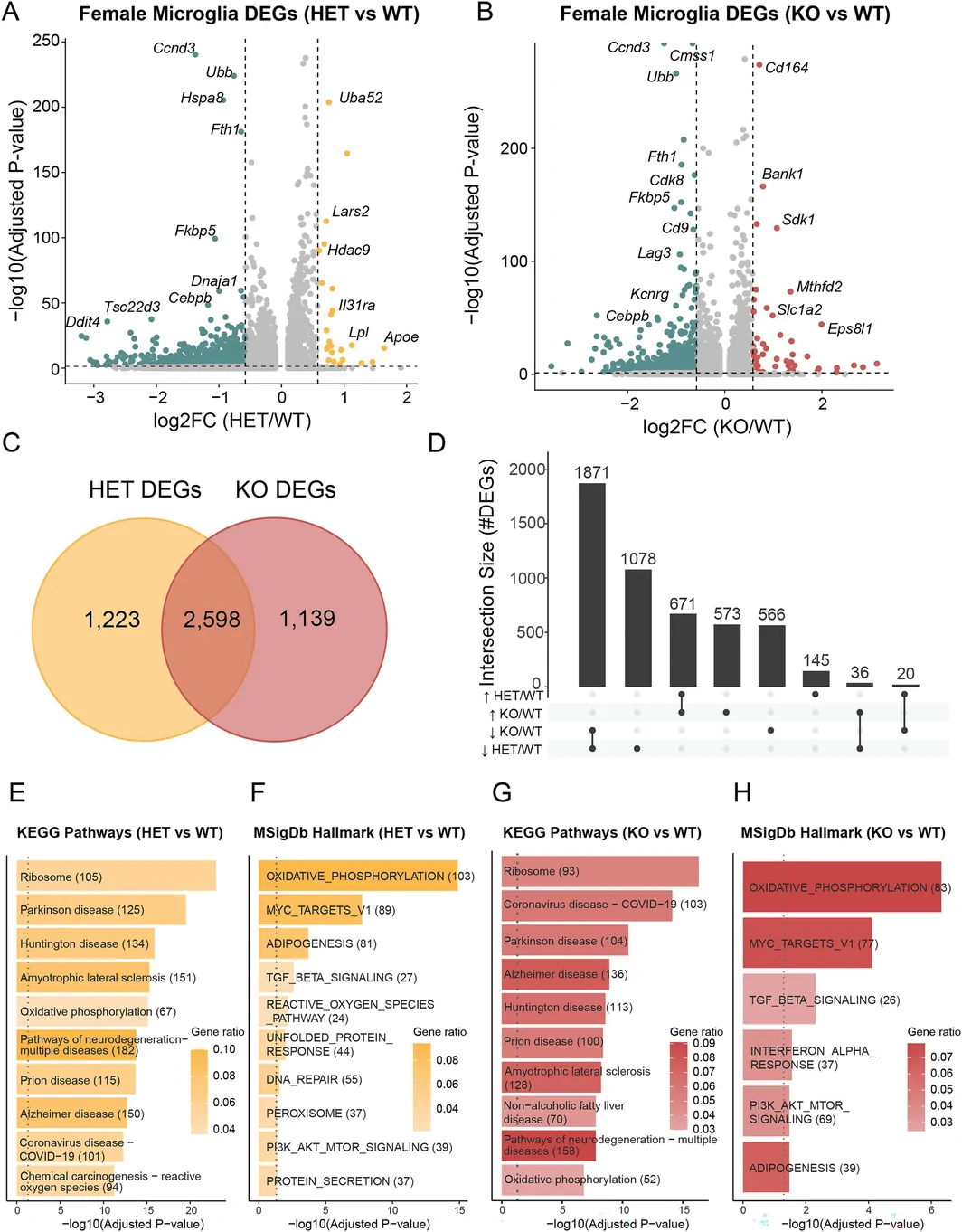
Cell‐level gene expression and pathway analyses of female microglia following *Plcg2* loss. (A, B) Volcano plots showing differentially expressed genes (DEGs) in female microglia comparing *Plcg2* HET versus WT (A) and KO versus WT (B). Genes meeting significance criteria (Bonferroni adjusted *p* value < 0.05, |log2FC| > 0.58) are highlighted, with select genes labeled. (C) Venn diagram of cell‐level DEGs meeting the stated thresholds in HET and KO microglia. (D) UpSet plot visualizing the intersection sizes for up‐ and down‐regulated gene sets among HET and KO groups compared to WT. (E–H) Functional enrichment analyses of differentially expressed genes identified in *Plcg2* HET versus WT and KO versus WT microglia. (E, G) KEGG pathway enrichment analysis and (F, H) MSigDB Hallmark pathway enrichment analysis for HET versus WT (E, F) and KO versus WT (G, H) comparisons. The *x*‐axis represents enrichment significance as −log10(Benjamini Hochberg‐adjusted *p* value). Bar color indicates gene ratio, defined as the proportion of input genes overlapping with each pathway gene set. Numbers in parentheses indicate the number of overlapping genes contributing to each enriched pathway term. The dashed line represents statistical cutoff for significance (*p*‐adjusted < 0.05).

To determine the biological processes associated with these gene‐expression changes, we performed pathway enrichment analyses using KEGG and MSigDB Hallmark gene sets. In both HET and KO microglia, enriched pathways were strongly associated with ribosomal function, oxidative phosphorylation, mitochondrial metabolism, reactive oxygen species pathways, and signaling programs linked to cellular stress and neurodegeneration‐related processes (Figure 3E–H; Tables S20 and S21). Direct KO versus HET pathway analysis identified additional enrichment in inflammatory and stress‐response pathways, including interferon signaling, complement activation, and mTORC1 signaling (Figure S5C,D and Table S22). Overall, these findings indicate that partial and complete *Plcg2* loss disrupt largely overlapping pathway‐level expression patterns in female microglia, while complete loss of function produces additional genotype‐specific perturbations.

We next examined the effects of partial and complete *Plcg2* loss on male microglial gene‐expression patterns. Sample‐aware pseudobulk analysis identified genotype‐dependent pathway alterations despite relatively modest gene‐level effects (Figure S6A,D and Table S23). HET males showed negative enrichment of ribosomal and oxidative phosphorylation pathways and multiple neurodegenerative disease signatures, with Hallmark GSEA additionally identifying reduced metabolic, mTORC1, and inflammatory programs (Figure S6B,C and Table S24). KO males similarly showed negative enrichment of ribosomal pathways, together with reduced DNA replication and positive enrichment of NOD‐like receptor signaling (Figure S6E and Table S25).

To further characterize the breadth and concordance of these expression patterns, we performed exploratory cell‐level differential expression analyses comparing HET and KO mice to sex‐matched WT controls (Figure 4A,B and Table S26). In contrast to females, which showed similar transcriptomic changes in HET and KO microglia relative to WT, male microglia exhibited substantially greater cell‐level gene‐expression changes following complete *Plcg2* deletion compared to loss of one allele. Comparison of HET/WT and KO/WT differential expression profiles identified 414 shared DEGs, while 144 genes were unique to HET/WT comparison and 3588 genes were uniquely dysregulated in KO/WT microglia (Figure 4C). We next asked whether these shared DEGs changed in the same or opposite direction between genotypes, splitting each intersection by direction of change relative to WT in an UpSet plot (Figure 4D and Table S27). Similarly to females, the majority of shared DEGs (342/414, 82.6%) were concordantly regulated, whereas 72 genes (17.4%) were discordant between genotypes. This suggests that many *Plcg2*‐dependent transcriptomic changes are preserved between heterozygous and complete loss‐of‐function states despite the substantially expanded KO transcriptomic signature.

**FIGURE 4:**
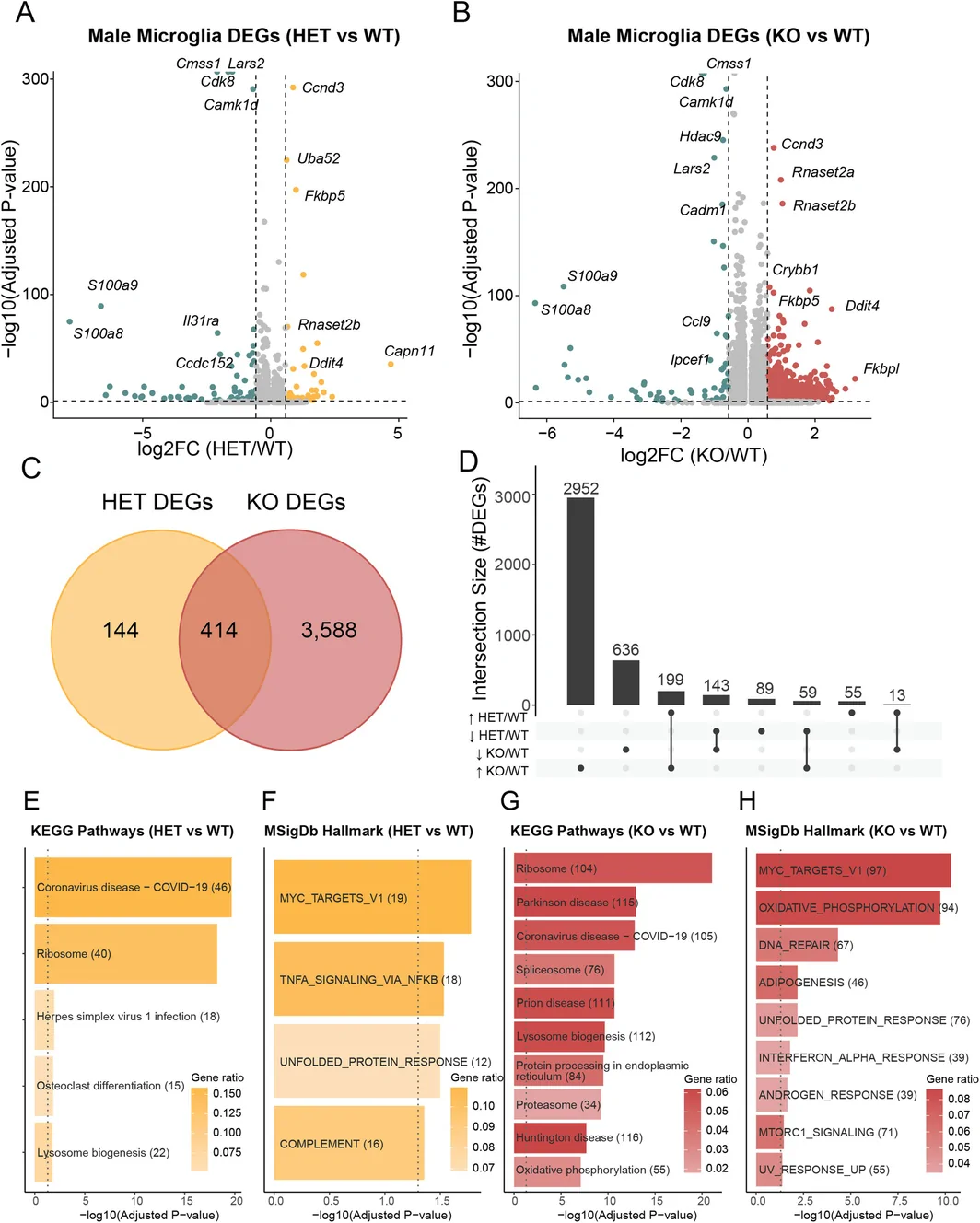
Cell‐level gene expression and pathway analyses of male microglia following *Plcg2* loss. (A, B) Volcano plots showing differentially expressed genes (DEGs) in male microglia comparing *Plcg2* HET versus WT (A) and KO versus WT (B). Genes meeting significance criteria (Bonferroni adjusted *p* value < 0.05, |log2FC| > 0.58) are highlighted, with select genes labeled. (C) Venn diagram of DEGs in HET and KO microglia. (D) UpSet plot visualizing the intersection sizes for up‐ and down‐regulated gene sets among HET and KO groups compared to WT. (E–H) Functional enrichment analyses of differentially expressed genes identified in *Plcg2* HET versus WT and KO versus WT microglia. (E, G) KEGG pathway enrichment analysis and (F, H) MSigDB Hallmark pathway enrichment analysis for HET versus WT (E, F) and KO versus WT (G, H) comparisons. The *x*‐axis represents enrichment significance as −log10(Benjamini Hochberg‐adjusted *p* value). Bar color indicates gene ratio, defined as the proportion of input genes overlapping with each pathway gene set. Numbers in parentheses indicate the number of overlapping genes contributing to each enriched pathway term. The dashed line represents statistical cutoff for significance (*p*‐adjusted < 0.05).

To further evaluate the relationship between HET‐ and KO‐associated gene expression changes compared to WT, we compared effect sizes for shared DEGs between the two comparisons. Shared genes exhibited strong concordance between HET and KO effect sizes (Pearson *r* = 0.904, *R* ^2^ = 0.817). Furthermore, linear regression analysis demonstrates that HET effect sizes closely approximate those observed in KO animals (slope = 0.945, 95% CI: 0.902–0.988; Figure S7A). We next assessed monotonic gene expression changes in male samples by identifying genes exhibiting concordant directional changes in both HET and KO animals, with greater effect magnitude in KO relative to HET comparisons. Using these criteria, we identified 207 monotonic genes in male microglia (Figure S7A and Table S28). Together, these findings indicate that heterozygous loss reproduces many *Plcg2*‐associated gene‐expression changes observed following complete deletion in males. However, the substantially larger set of KO‐specific DEGs suggests that complete *Plcg2* deletion induces broader transcriptomic changes beyond those observed in the heterozygous state. To more directly assess genotype‐dependent effects, we next performed differential expression analysis between male KO and HET microglia (Figure S7B and Table S26). Consistent with the expanded KO transcriptomic profile, direct genotype comparison identified substantial expression profile divergence between heterozygous and complete loss‐of‐function states in males.

Pathway enrichment analysis revealed both shared and genotype‐specific biological processes associated with *Plcg2* perturbation in males. HET microglia exhibited enrichment for ribosomal, lysosomal, complement, and inflammatory signaling pathways (Figure 4E,F and Table S29). In contrast, KO microglia demonstrated broader enrichment across pathways related to oxidative phosphorylation, proteostasis, RNA processing, mitochondrial function, stress‐response signaling, and neurodegeneration‐associated processes (Figure 4G,H and Table S30). Direct KO versus HET enrichment analysis further highlighted pathways associated with oxidative phosphorylation, unfolded protein response, interferon signaling, and neurodegeneration‐related annotations (Figure S7C,D and Table S31). Together, these findings indicate that complete *Plcg2* loss produces a broader gene‐expression signature change in male microglia than heterozygous loss, while preserving a core set of shared *Plcg2*‐associated expression changes.

### 3.4 Opposing Directions of Expression Change in Male and Female *Plcg2*‐Deficient Microglia Converge on Neurodegeneration‐Related Pathways

Our sample‐level pseudobulk analyses suggested that sex may influence the gene‐expression consequences of *Plcg2* perturbation (Figure 2 and Figure S3). Variance partitioning identified sex‐by‐genotype effects as an important source of gene expression variability (Figure 2G). Furthermore, interaction GSEA identified coordinated pathway‐level enrichment for biologically and disease‐relevant terms (Figure 2H). However, no individual genes reached FDR significance for the interaction term itself, and the limited sample size restricts our ability to exclude more moderate interaction effects. We therefore examined our sex‐stratified cell‐level analyses as a complementary approach for identifying candidate expression patterns. We emphasize that differences between sex‐stratified estimates do not themselves constitute a statistically significant genotype‐by‐sex interaction. Rather, this comparison is intended to identify gene‐expression patterns that may underlie the pathway‐level interaction signals identified above and that warrant further targeted investigation.

With this comparative framework in place, we first compared pathway enrichment among HET/WT and KO/WT transcriptomic signatures from females and males. Many of the most strongly enriched pathways were those associated with neurodegenerative diseases, with Parkinson disease (mmu05012), Prion disease (mmu05020), and Huntington disease (mmu05016) among the most significantly enriched programs across female HET, female KO, and male KO microglia relative to sex‐matched WT controls (Figure 5A). These results were observed despite these mice being young and lacking any neurodegenerative pathology. Similar enrichment patterns were also observed using the MSigDB Hallmark gene set annotations, including pathways related to oxidative phosphorylation, proteasomal degradation, inflammatory signaling, and receptor‐mediated signaling cascades (Figure S8). In contrast, male HET microglia exhibited comparatively fewer enrichment terms, consistent with the smaller number of DEGs identified in this group (Figure 4C).

**FIGURE 5:**
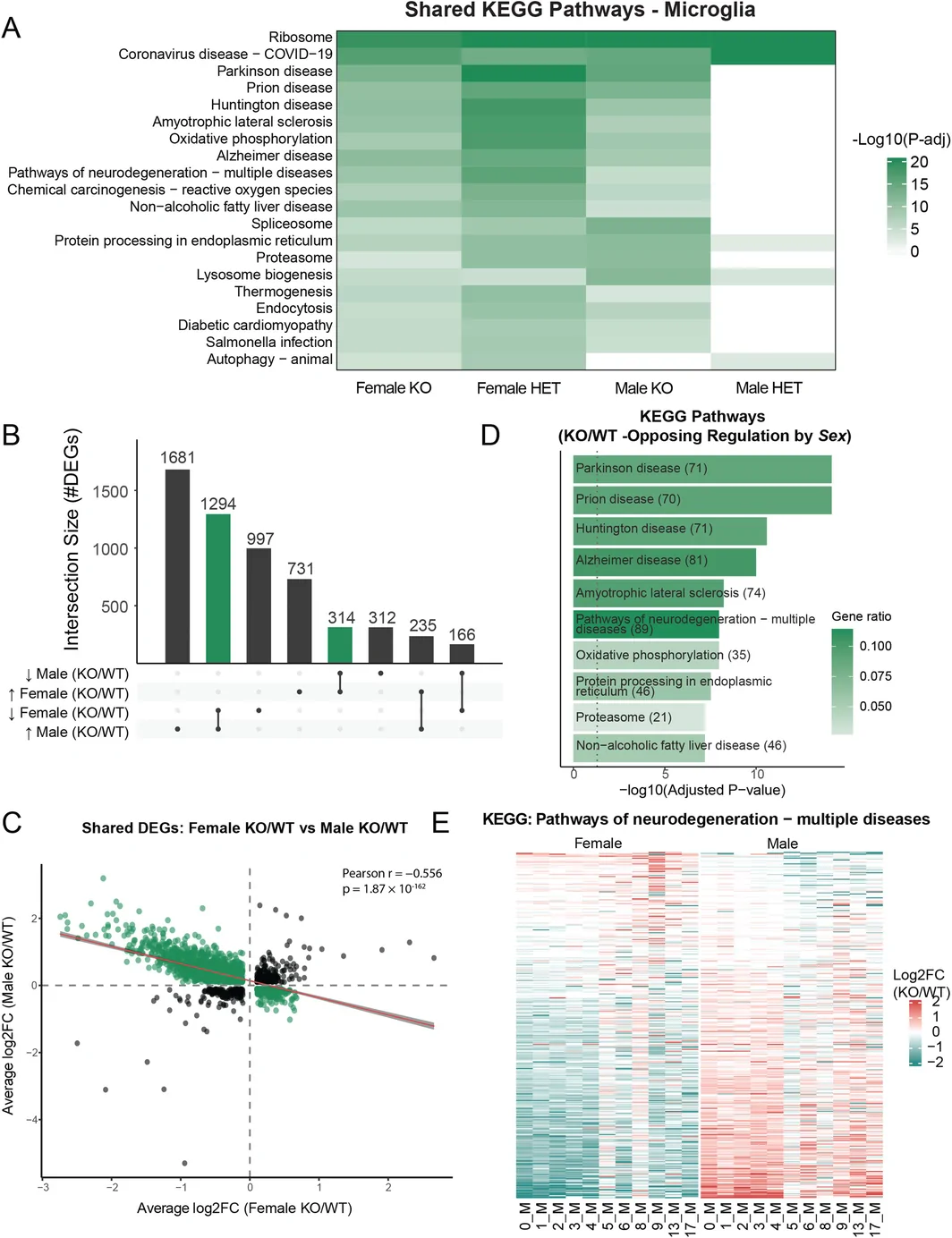
Sex‐stratified analysis of divergent microglial gene‐expression and pathway patterns following *Plcg2* loss. (A) Heatmap showing significant enrichment for top KEGG disease and cellular pathways among differentially expressed genes (DEGs) in KO and HET microglia compared to sex‐matched WT microglia. Terms are sorted and colored by −log10(Benjamini Hochberg‐adjusted *p* value), with white indicating no significance for that comparison. (B) UpSet plot illustrating intersection sizes for upregulated and downregulated DEGs in male and female KO conditions compared to WT. Comparisons showing opposite directionality between sexes are highlighted in green. (C) Scatter plot showing shared cell‐level DEGs between female KO/WT and male KO/WT microglia. Each point represents a shared DEG, plotted by average log2 fold change (log2FC) in female KO/WT (*x*‐axis) and male KO/WT (*y*‐axis) comparisons. Genes exhibiting opposite fold‐change directions between sexes are highlighted in green. Shaded area around the regression line represents the 95% confidence interval. (D) Bar plot depicting the most significantly enriched KEGG pathways among microglial DEGs showing opposite fold‐change directions between female and male KO groups. The *x*‐axis represents enrichment significance as −log10(Benjamini Hochberg‐adjusted *p* value). Bar color indicates gene ratio, defined as the proportion of input genes overlapping with each pathway gene set. Numbers in parentheses indicate the number of overlapping genes contributing to each enriched pathway term. (E) Heatmap of log2 fold change values in genes from the “pathways of neurodegeneration − multiple diseases” KEGG term (mmu05022) across microglial clusters, comparing microglial subclusters in female and male KO mice versus WT. Red indicates positive KO‐versus‐WT log2 fold change, and teal indicates negative KO‐versus‐WT log2 fold change.

To examine the directionality of the sex‐stratified responses underlying these shared pathway annotations, we next compared DEGs identified independently in male and female KO/WT analyses. Genes were classified as exhibiting “opposite” regulation if they met predefined differential expression thresholds in both sex‐stratified comparisons (Bonferroni‐adjusted *p* < 0.05, |log2FC| > 0.1) but displayed opposing fold‐change directions relative to their respective sex‐matched WT controls. Although many DEGs were unique to individual groups, the subset of genes that overlapped across groups showed strikingly directional discordance in KO mice relative to sex‐matched wild‐type controls (Table S32). For example, 1294 genes were upregulated in male KO but downregulated in female KO microglia and 314 genes were downregulated in male KO but upregulated in female KO microglia (Figure 5B). In contrast, only 235 genes were upregulated in both male and female KO microglia and 166 genes were downregulated in both male and female KO microglia (Figure S9A). Similar directional discordance was also observed between female and male HET microglia, although the magnitude of overlap was substantially reduced (Figure S9C,E). Overall, these analyses revealed that, among DEGs significant in both male KO/WT and female KO/WT microglial comparisons, approximately 80% of genes meeting DEG criteria showed opposite directions of change between sexes.

To further quantify these directional differences in gene expression, we compared log2 fold change values for DEGs shared between female and male KO microglia. Shared DEGs demonstrated an inverse relationship in effect sizes between male and female (Pearson *r* = −0.556, *p* = 1.87 × 10^−162^), indicating that genes upregulated in one sex tended to be downregulated in the other (Figure 5C). Linear regression yielded a slope of −0.606 (95% CI, −0.645 to −0.566), further supporting this directional discordance. Together, these exploratory sex‐stratified analyses revealed pronounced directional discordance in the gene‐expression patterns associated with *Plcg2* loss. These findings are consistent with the pathway‐level genotype‐by‐sex interaction signals identified above (Figure 2H and Figure S3).

To determine whether these discordant gene‐expression patterns converged on specific biological processes, we performed pathway enrichment analysis using only oppositely regulated DEGs. This analysis identified enrichment for KEGG pathways associated with many neurodegenerative diseases including Parkinson disease (mmu05012), Huntington disease (mmu05016), Alzheimer disease (mmu05010), and the KEGG “Pathways of neurodegeneration – multiple diseases” annotation (mmu05022; Figure 5D and Table S33). However, this enrichment of neurodegenerative terms was not observed in shared DEGs going in the same direction (Figure S9B and Table S33). Enrichment of neurodegenerative terms was also not observed from any overlapping DEGs between male and female HET microglia (Figure S9D,F and Table S33). Genes contributing to the KEGG ‘Pathways of neurodegeneration’ enrichment were associated with proteostasis, mitochondrial function, and inflammatory/stress signaling, suggesting broad dysregulation of multiple glial processes implicated in neurodegenerative disease (Figure S10). While membership in this broad KEGG annotation does not by itself establish a direct mechanistic relationship between each constituent gene and neurodegeneration, the representation of these cellular functions provides biological context for the enrichment.

To visualize the directionality of expression changes across the broader pathway annotation, we next examined KO‐versus‐WT fold changes for expressed genes within the complete mmu05022 gene set across microglial clusters (Figure 5E and Table S34). Overall, this visualization shows widespread, directionally opposing expression changes across the neurodegeneration‐associated gene set. This consistency indicates that the pathway‐level signal reflects a coordinated, sex‐associated shift in the cellular programs represented within mmu05022, rather than an effect attributable to only a limited subset of genes or clusters.

### 3.5 Mice Lacking *Plcg2* Show Sex‐Associated Changes in Microglia and Astrocytes at the System Level

While DEG analysis identifies individual genes with significant expression changes, it does not fully capture broader patterns of coordinated gene regulation. To complement these analyses, we applied high‐dimensional Weighted Gene Co‐expression Network Analysis (hdWGCNA) to our single‐cell dataset. Rather than focusing on differential expression alone, hdWGCNA identifies modules of co‐expressed genes based on correlation structure across cells, enabling the identification of coordinated expression patterns and the functional annotation of cell type–associated gene modules. Module construction and eigengene calculation in hdWGCNA incorporate sample identity (via metacell aggregation within samples), helping to mitigate sample‐driven artifacts at the network‐building stage. This systems‐level approach therefore provides information distinct from, and complementary to, conventional DEG analysis. Using this approach, we identified four distinct co‐expression modules in our dataset (Figure S11A and Table S35). Modules 1, 2, and 3 were expressed predominantly in microglia, whereas Module 4 was expressed primarily in astrocytes (Figure 6A and Figure S11B). Notably, Modules 2 and 3 showed highest expression in the *Plcg2*‐high microglial clusters 5_M and 8_M (Figure S11C).

**FIGURE 6:**
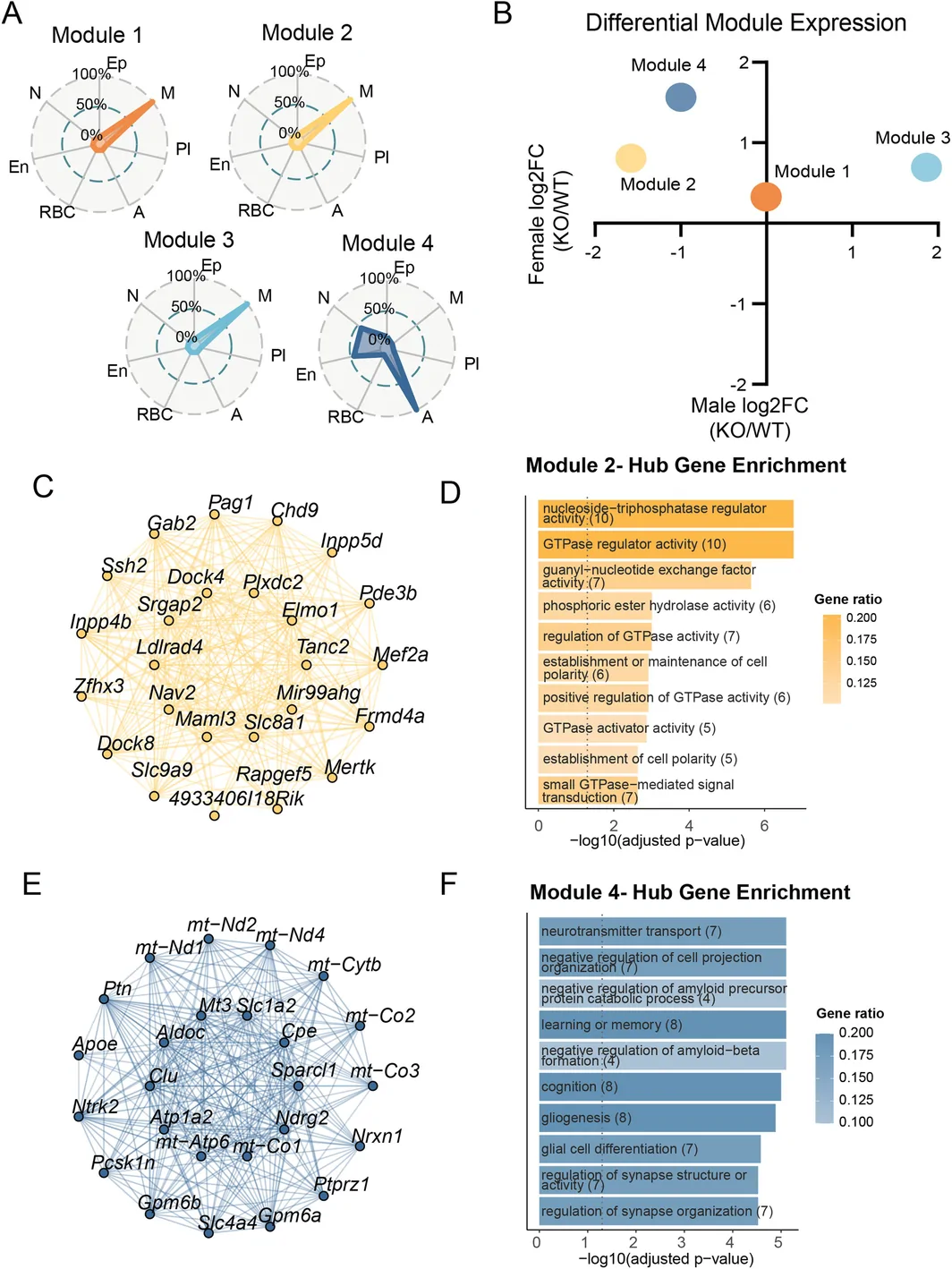
Module‐level network analysis of gene co‐expression reveals sex‐associated module regulation across glial cell types. (A) Radar plots showing cell type specificity for four co‐expression modules identified by hdWGCNA. Expression showed as average module eigengene as scaled expression across cell types. (B) Scatter plot depicting differential module expression (log2 fold change KO/WT) in male versus female cells for each module. Modules in the upper‐left and lower‐right quadrants show opposite KO‐versus‐WT fold‐change directions in males and females. (C, E) Network visualizations showing top 25 hub genes identified within Module 2 (C) and Module 4 (E). Nodes represent hub genes and edge opacity represents co‐expression weights within each module. (D, F) Bar plots displaying significantly enriched Gene Ontology (GO) associated with hub genes from Module 2 (D) and Module 4 (F). The *x*‐axis represents enrichment significance as −log10(Benjamini Hochberg‐adjusted *p* value). Bar color indicates gene ratio, defined as the proportion of hub genes overlapping with each GO term. Numbers in parentheses indicate the number of overlapping genes contributing to each enriched GO process. The dashed line represents the statistical cutoff for significance (adjusted‐*p* < 0.05).

Given the sex‐driven divergence observed in DEG and enrichment analyses (Figure 5B,C), we examined differential module eigengene (DME) analysis between KO and WT males and females (Table S36). Notably, in separate sex‐stratified KO‐versus‐WT comparisons, Modules 2 and 4 showed eigengene shifts in opposite directions in males and females (Figure 6B). Module‐trait correlation analysis identified associations between module eigengenes and both genotype (Plcg2_HET, Plcg2_KO) and sex (sex_M) across nearly all cell types (Figure S12). Microglia, astrocytes, and peripheral immune cells also showed associations with combined sex‐and‐genotype categories (male‐HET, male‐KO) for Modules 2 and 4, with these modules showing predominantly lower eigengene expression in cells from male HET and KO mice relative to the other sex‐genotype combinations (FDR‐corrected *p* < 0.05) (Figure S12). The module‐trait correlation statistics reported here were computed at the individual‐cell level rather than explicitly modeling sample as the unit of replication and should therefore be interpreted primarily as descriptive of cell‐level trends within this dataset. However, these module patterns were consistent with the directional differences observed in the exploratory sex‐stratified DEG analyses (Figure 5B,C) and identify candidate module‐level patterns for testing in larger, replicate‐aware cohorts.

To better understand the biological functions of these modules, we next performed enrichment analyses on the top module member genes ranked by eigengene connectivity (kME), using the top 25 genes for module network visualization and the top 50 genes for enrichment analyses (Table S37). Module 2, which was expressed primarily in microglia, contained hub genes associated with immune signaling and cytoskeletal regulation and was enriched for pathways related to GTPase activity and cell polarity (Figure 6C,D). Module 1 was more highly expressed in homeostatic and inflammatory microglia and enriched for ribosomal genes (Figure S11D), whereas Module 3 was only nominally enriched for developmental pathways (Figure S11E).

In contrast, Module 4 represented an astrocyte‐enriched co‐expression network comprising 236 genes (Table S35). This module included astrocyte‐associated genes such as *Apoe* and *Clu* and was enriched for pathways related to amyloid‐beta regulation and cognition (Figure 6E,F). Notably, Module 4 also exhibited strong sex‐associated eigengene behavior, with opposite directional shifts observed between male and female KO mice in sex‐stratified analysis. Together, these findings suggest that Module 4 may capture an astrocyte‐associated expression pattern that differs across sex‐genotype groups.

### 3.6 Sex‐Stratified Analyses Identify Divergent Gene‐Expression Patterns in *Plcg2*‐Deficient Astrocytes

Because hdWGCNA identified a distinct astrocyte‐enriched module with sex‐associated differences in module eigengene expression (Figure 6A,B and Figure S11B), we next examined whether *Plcg2* perturbation produced corresponding genotype‐ and sex‐associated changes in the astrocyte transcriptome. Mouse‐level pseudobulk analysis identified genotype‐associated pathway changes in astrocytes after accounting for sex and technical batch (Figure S13A–D and Table S38). HET astrocytes showed prominent negative enrichment of ribosomal, oxidative phosphorylation, and multiple neurodegenerative disease‐associated pathways, whereas KO astrocytes showed a distinct profile characterized primarily by reduced immune‐, cytoskeletal‐, and signaling‐associated pathways (Figure S13B,D and Table S39). Formal genotype‐by‐sex interaction GSEA further identified significant enrichment of gene sets ranked by the interaction statistic for both HET and KO comparisons, including metabolic, ribosomal, and neurodegeneration‐associated gene sets (Figure S13E,F; Tables S40 and S41). Sex‐stratified mouse‐level analyses further revealed divergent pathway patterns between female and male astrocytes (Figure S14; Tables S42 and S43). Female KO astrocytes showed predominantly positive enrichment of oxidative phosphorylation, ribosomal, and neurodegeneration‐associated pathways, whereas the corresponding pathways were negatively enriched in male KO astrocytes. Male KO astrocytes instead showed reduced enrichment of several immune‐ and cytoskeletal‐associated pathways. Together, these sample‐aware analyses indicate that *Plcg2* perturbation is linked with sex‐associated differences in coordinated astrocyte pathway patterns and motivated further examination of the directionality of these patterns at the cell level.

To further determine the gene‐level patterns underlying these pathway‐level differences, we performed complementary exploratory cell‐level analyses comparing HET and KO astrocytes with sex‐matched WT controls. Differential expression analysis revealed divergent cell‐level gene‐expression patterns between male and female astrocytes following *Plcg2* deletion (Figure 7A,B and Table S44). To determine whether partial *Plcg2* loss produced similar astrocytic effects, we additionally analyzed differential expression between HET and WT astrocytes (Figure S15A,B and Table S44). Consistent with the microglial analyses, female HET astrocytes exhibited a broader cell‐level DEG signature than male HET astrocytes.

**FIGURE 7:**
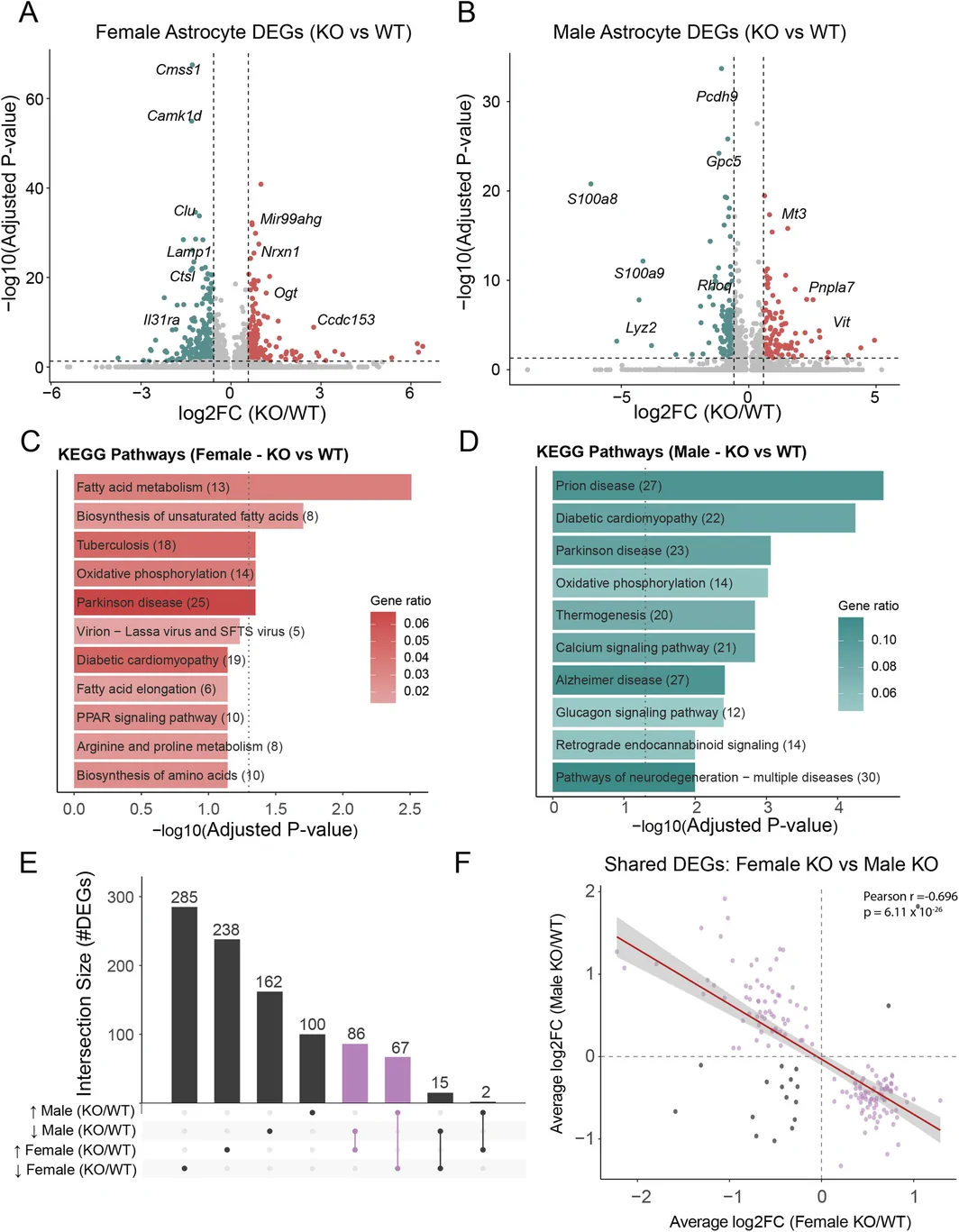
Cell‐level analysis of sex‐stratified astrocyte gene‐expression and pathway patterns following *Plcg2* loss. (A, B) Volcano plots showing differentially expressed genes (DEGs) comparing KO/WT in female (A) or male (B) astrocytes. (C, D) KEGG pathway enrichment analyses of DEGs identified in female (C) and male (D) astrocytes comparing KO/WT conditions. The *x*‐axis represents enrichment significance as −log10(Benjamini Hochberg‐adjusted *p* value). Bar color indicates gene ratio, defined as the proportion of input genes overlapping with each pathway gene set. Numbers in parentheses indicate the number of overlapping genes contributing to each enriched pathway term. Female astrocyte DEGs were enriched for lipid metabolism and oxidative phosphorylation pathways, whereas male astrocyte DEGs were enriched for neurodegenerative disease‐associated and mitochondrial signaling pathways. The dashed line represents the statistical cutoff for significance (adjusted *p* value < 0.05). (E) UpSet plot illustrating intersection sizes for upregulated and downregulated DEGs in male and female KO versus WT astrocytes. Comparisons showing opposite directionality between sexes are highlighted in purple. (F) Scatter plot showing shared DEGs between female KO/WT and male KO/WT astrocytes. Each point represents a shared DEG, plotted by average log2 fold change (log2FC) in female KO/WT (*x*‐axis) and male KO/WT (*y*‐axis) comparisons. Genes with opposite fold‐change directions between sexes are highlighted in purple, with the regression line illustrating the overall relationship between male and female astrocyte gene‐expression patterns following *Plcg2* loss. Shaded area around the line represents the 95% confidence interval.

KEGG pathway enrichment analysis of female KO astrocytes identified pathways related to fatty acid metabolism, biosynthesis of unsaturated fatty acids, as well as Parkinson disease (Figure 7C and Table S45). Male KO astrocytes exhibited enrichment for pathways associated with calcium signaling as well as more enrichment for KEGG pathways associated with neurodegenerative diseases, including Alzheimer disease (mmu05010), Parkinson disease (mmu05012), Prion disease (mmu05020), and “Pathways of neurodegeneration – multiple diseases” (mmu05022, Figure 7D and Table S45). These findings indicate that *Plcg2* loss induces distinct metabolic, signaling, and neurodegeneration‐associated pathway patterns in astrocytes. Pathway enrichment analysis across all astrocyte comparisons identified shared enrichment for Parkinson disease between female KO/WT, female HET/WT, and male KO/WT DEGs (Figure S15C; Tables S45 and S46). Male HET/WT astrocyte DEGs exhibited minimal pathway enrichment, consistent with the smaller number of DEGs identified in this comparison.

We next compared DEG directionality between female and male KO astrocytes to determine whether astrocytes exhibited a pattern of directional discordance similar to that observed in microglia. Many DEGs were unique to one sex, while overlapping gene sets frequently exhibited opposing directions of change between male and female KO astrocytes compared to sex‐matched WT samples (Figure 7E). Specifically, 67 genes were downregulated in female KO astrocytes but upregulated in male KO astrocytes, and 86 genes showed the reciprocal pattern, being upregulated in female KO and downregulated in male KO astrocytes. In contrast, substantially fewer genes were changed concordantly across sexes, with 15 genes upregulated and 2 genes downregulated in both male and female KO astrocytes (Figure 7E). Overall, approximately 90% of genes (153/170) meeting DEG criteria in both male KO/WT and female KO/WT comparisons showed opposite fold‐change directions between sexes (Figure 7E).

This discordance was also evident in the magnitude of the effects. Across the same shared DEGs, male and female log2 fold changes demonstrated a strong inverse relationship (Pearson *r* = −0.696, *p* = 6.11 *x* 10^−26^). Linear regression yielded a slope of −0.725 (95% CI −0.839 to −0.611), further supporting pronounced directional discordance between the two sex‐stratified comparisons (Figure 7F). Because relatively few oppositely changed astrocyte DEGs were shared between sexes, pathway enrichment analysis identified only nominally significant KEGG terms. Despite this limitation, the enriched pathways again included pathways associated with neurodegenerative diseases such as Alzheimer disease and Prion disease (Tables S47 and S48). Together, these exploratory analyses indicate that astrocytes, like microglia, exhibit sex‐divergent gene‐expression patterns following *Plcg2* loss, extending the directional differences observed in microglia to an additional glial population.

### 3.7 Loss of *Plcg2* May Alter Signaling From Microglia to Other Cell Types Differentially in Male and Female Mice

Next, we sought to determine whether *Plcg2* deletion was associated with alterations in predicted intercellular communication patterns involving microglia and other cell populations in our dataset. To investigate sex‐stratified differences in intercellular communication, we applied CellChat to our single‐cell transcriptomic data from female and male mice (Table S49), identifying putative ligand‐receptor interactions that provide a hypothesis‐generating framework for understanding how *Plcg2* loss may reshape intercellular communication.

In female KO mice, CellChat identified 954 predicted ligand–receptor interaction pairs compared with 489 in WT controls, indicating greater inferred network connectivity in the KO condition (Figure 8A). The global signaling network visualization showed higher predicted microglia‐associated communication probabilities with most cell types in female KO mice, while predicted interactions with peripheral immune populations were reduced (Figure 8B). Chord diagrams showed higher predicted microglia–astrocyte communication involving pathways such as APP and cholesterol signaling (Figure 8C). In contrast, predicted immune‐related and adhesion‐associated pathways, including DHEAS, CCL, and CD39 signaling, showed lower communication probabilities in KO females (Figure 8D). Visualization of the underlying microglia–astrocyte ligand–receptor networks similarly showed broad differences in predicted communication patterns in female KO mice (Figure S16A).

**FIGURE 8:**
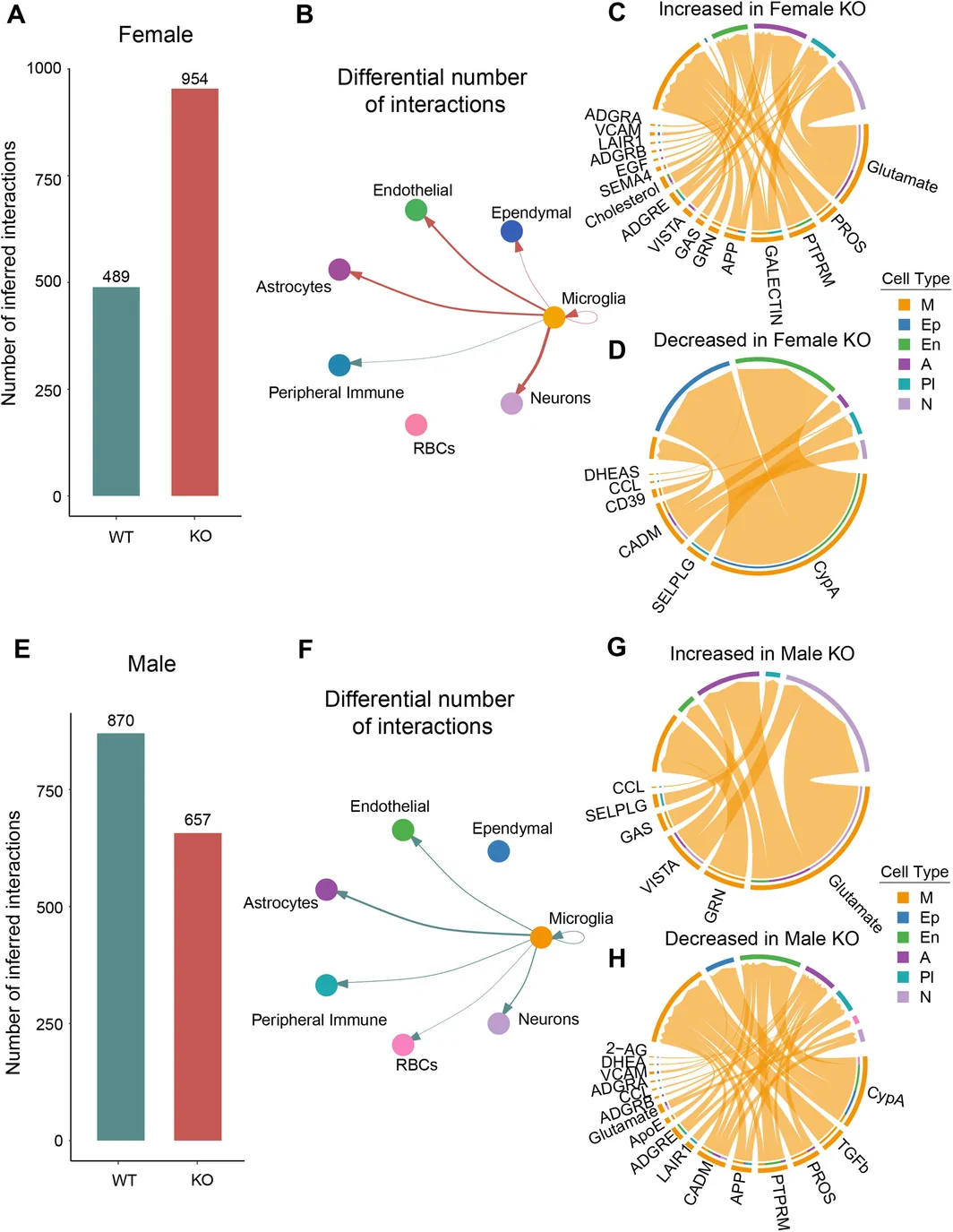
CellChat predicts sex‐stratified differences in cell–cell communication following *Plcg2* loss. (A) Bar plots showing the total number of predicted cell–cell interactions in female WT and KO brains, as inferred by CellChat. (B) Network graphs depicting KO‐versus‐WT differences in predicted interaction frequencies between microglia and other major cell types in female samples. Edge thickness reflects the strength of interaction as measured by computed communication probability. Red lines indicate higher predicted communication probability in KO, whereas blue indicates lower predicted communication probability in KO. (C, D) Chord diagrams representing KO‐versus‐WT differences in predicted outgoing interactions from microglia to other cell types in female samples. (C) Predicted interactions higher in KO. (D) Predicted interactions lower in KO. Plot circumference shows outgoing signaling from cell type (outer band) to recipient cell type (inner band). The width of the band (or edge) represents interaction probability. (E–H) Bar plot (E), network graph (F), and chord diagram (G, H) for male samples as described above.

By contrast, CellChat inferred fewer ligand–receptor interaction pairs across cell populations in male KO mice relative to WT mice (Figure 8E). The male network showed lower overall predicted connectivity, with reduced communication probabilities involving microglia across most cell types (Figure 8F). Predicted interactions that were higher in male KO mice included CCL, SELPLG, and VISTA signaling (Figure 8G), whereas APP‐ and ApoE‐associated communication probabilities were lower (Figure 8H). Notably, some pathways, including APP‐associated signaling, showed lower predicted communication in male KO mice but higher predicted communication in female KO mice (Figure 8C,H). Visualization of the corresponding microglia–astrocyte ligand–receptor networks likewise showed broadly lower predicted connectivity in male KO mice relative to WT controls (Figure S16B).

Together, these findings identify sex‐associated differences in predicted intercellular communication networks following *Plcg2* loss under physiological conditions. Future studies will be required to determine the functional significance and biological directionality of these predicted interactions.

## 4 Discussion

Previous research has primarily examined *Plcg2* function in the context of AD‐associated variants or under pathological conditions (Turner et al. 2026; Kleineidam et al. 2020; Claes et al. 2022; Diks et al. 2023; Magno et al. 2019; Takalo et al. 2020; Tsai et al. 2023; Li et al. 2022; Oblak et al. 2022). Consequently, the physiological role of *Plcg2* in the brain remains largely unexplored. As *Plcg2* emerges as a potential immunomodulatory target for AD, defining its functions across different biological contexts is critical. In this study, we investigated the impact of *Plcg2* deletion on glial gene‐expression profiles in the brains of young, non‐disease mice using single‐cell transcriptomics. Mouse‐level pseudobulk analyses identified *Plcg2*‐associated gene‐expression changes in microglia and astrocytes, while GSEA of the formal genotype‐by‐sex interaction statistic revealed coordinated enrichment of metabolic, immune, and neurodegeneration‐associated pathways despite the absence of FDR‐significant individual‐gene interactions (Figure 2; Figures S3 and S13). Complementary sex‐stratified analyses identified pronounced directional differences in *Plcg2*‐associated expression patterns between males and females, providing additional biological context for these pathway‐level interaction signals while remaining distinct from a formal test of interaction (Figure 5). We additionally observed alterations in glial co‐expression and predicted intercellular communication networks (Figures 6 and 8). Furthermore, to our knowledge, this is the first study to determine the effects of *Plcg2* deletion on transcriptomic changes in astrocytes at the single‐cell level (Figure 7). Together, these findings define baseline gene expression consequences associated with *Plcg2* deficiency and provide a resource for investigating PLCG2 biology across genotype and sex.

Co‐expression and pathway‐enrichment analyses support a relationship between *Plcg2* expression and microglial programs involved in surveillance and cytoskeletal function under physiological conditions. *Plcg2* is co‐expressed with several AD‐associated genes that regulate cytoskeletal dynamics and motility, including *Blnk* and *Rhoh* (Figure 1E). PLCG2 and BLNK have an established functional relationship in B‐cell receptor signaling, where phosphorylated BLNK acts as a scaffold that recruits PLCG2 to the receptor‐associated signaling complex and facilitates its tyrosine phosphorylation and activation (Ishiai, Kurosaki, et al. 1999; Ishiai, Sugawara, et al. 1999; Fu et al. 1998). In contrast, co‐expression with genes such as *Rhoh* identifies candidate relationships whose functional connection to PLCG2 in microglia remains to be established. High *Plcg2‐*expressing clusters (5_M and 8_M) were enriched for pathways related to actin remodeling and phagocytosis (Figure S2C,D). Notably, Cluster 5_M was significantly reduced in female HET mice relative to WT controls, raising the possibility that partial *Plcg2* loss alters microglial subpopulations associated with surveillance and cytoskeletal regulation. The broader exploratory cell‐level expression patterns observed in female HET microglia raise the possibility that changes within this population contribute to the female expression signature, although this relationship will require confirmation at the biological‐replicate level. Together, these findings suggest a role for *Plcg2* in microglial surveillance even in the absence of pathological inflammation. This interpretation aligns with previous evidence that PLCG2 contributes to motility and phagocytic functions through calcium signaling and cytoskeletal reorganization (Obst et al. 2021; Solomon et al. 2022). Specifically, PLCG2 interacts with Rho GTPases, including RAC1, which drive lamellipodia formation and phagocytic cup assembly (Walliser et al. 2015; Rougerie et al. 2013).

*Plcg2* is often studied as a canonical downstream effector of TREM2 signaling as well as a regulator of *Trem2*‐independent processes (Magno et al. 2021; Messenger et al. 2025). In the TREM2‐dependent axis, ligand engagement drives DAP12 phosphorylation, recruitment and activation of SYK, and downstream activation of PLCG2. This process promotes calcium flux, cytoskeletal remodeling, and ultimately phagocytic clearance of lipid‐laden or amyloid substrates (Andreone et al. 2020; Obst et al. 2021; Wang et al. 2022; Aki et al. 2008). However, PLCG2 can also be activated downstream of other receptors expressed on microglia, including integrins and pattern recognition receptors, enabling potential TREM2‐independent modulation of similar effector programs (Li et al. 2022; Andreone et al. 2020; Jakus et al. 2009). *Plcg2* expression showed limited co‐expression with *Trem2* in high *Plcg2* expressing clusters (8_M and 5_M) (Figure 1E). This pattern raises the possibility that TREM2‐independent PLCG2‐associated pathways contribute to the surveillance‐associated expression profiles observed under baseline conditions, whereas TREM2‐dependent functions may become more prominent in pathological contexts. These findings highlight the need to consider *Plcg2* as a potential regulator of microglial surveillance in the healthy brain and open additional avenues by which *Plcg2* may modulate AD pathology.

Sex is an important determinant of microglial gene expression and function across development and adulthood (Bishnoi and Bordt 2025; Barko et al. 2022; Lenz and McCarthy 2015). Male microglia generally exhibit more reactive and immune‐active states, whereas female microglia show stronger enrichment for pathways associated with repair and inflammatory regulation (Villa et al. 2018; Hanamsagar et al. 2017; Guneykaya et al. 2018). These baseline differences in microglial functions between sexes may in part reflect distinct transcriptomic landscapes shaped by sex chromosomes and gonadal hormone exposure (Ocañas et al. 2023; Bobotis et al. 2025). As a result, the same genetic perturbation, such as *Plcg2* deletion, may engage distinct molecular programs in males and females rather than producing equivalent downstream responses. Furthermore, estrogen receptor signaling has broad immunomodulatory effects in myeloid cells that converge on calcium‐dependent pathways (Liu et al. 2013; Kovats 2015). Together, these observations raise the possibility that sex‐associated hormonal context may shape the functional consequences of PLCG2 perturbation. Consistent with this possibility, our formal interaction analysis identified pathway‐level enrichment associated with the genotype‐by‐sex interaction statistic, while exploratory sex‐stratified analyses revealed substantial directional discordance in gene‐expression patterns following *Plcg2* loss (Figures 2H and 5). Importantly, because no individual genes reached FDR significance for the interaction term, these data do not establish individual sex‐dependent genes; rather, they identify coordinated pathway‐level and sex‐stratified patterns that warrant testing in larger cohorts. Sex‐associated effects of PLCG2 have also been reported in other experimental contexts. Specifically, a recent pre‐print showed sex‐specific lipidomic changes in *Plcg2* KO mice (Hopp et al. 2023) and the protective *Plcg2* ^*P522R*^ variant shows a similar sexually divergent effect in peripheral macrophages (Staley et al. 2024). Other AD risk genes, including *Inpp5d*, have likewise shown sex‐associated microglial expression differences (Dabin et al. 2024), and are co‐expressed with *Plcg2* within our microglial populations (Figure 1E). Together, these observations suggest that sex‐associated differences in baseline microglial biology may shape the transcriptomic consequences of *Plcg2* deficiency, providing a biological framework for the divergent pathway‐level responses observed between males and females.

Additionally, comparison of HET and KO animals suggests that the transcriptomic consequences of *Plcg2* loss are not uniformly proportional to gene dosage. In the mouse‐level comparisons, the sex‐adjusted KO versus WT analysis identified about 100 significant DEGs while only one gene reached significance for the HET versus WT analysis (Figure 2). In the complementary cell‐level analyses, however, HET‐ and KO‐associated effect sizes were strongly concordant among shared genes in both sexes, indicating overlap between the expression patterns associated with partial and complete *Plcg2* loss. However, this relationship differed between sexes. Female HET microglia more closely resembled female KO microglia, whereas complete deletion in males was associated with a substantially broader gene‐expression signature than heterozygous loss. Direct HET versus KO comparisons and monotonic analyses similarly identified both shared and genotype‐specific responses. Because the strongest HET–KO similarities were observed in exploratory cell‐level analyses, we interpret these findings as evidence for potentially overlapping consequences of partial and complete *Plcg2* loss rather than as establishing a haploinsufficiency‐like effect. Larger mouse‐level cohorts will be needed to determine whether these patterns reflect true dosage‐dependent biology. Given that human *PLCG2* variants are predominantly heterozygous, these findings may have important implications for understanding how partial perturbation of PLCG2 function influences glial biology in human disease.

Several analyses repeatedly identified pathways annotated by KEGG for Alzheimer's disease, Parkinson's disease, Huntington's disease, prion disease, and other neurodegenerative disorders. These findings require cautious interpretation because the mice studied here were young and lacked amyloid, tau, or other overt neurodegenerative pathology. Many genes underlying these broad annotations participate in fundamental cellular processes, including mitochondrial function, oxidative phosphorylation, proteostasis, ribosomal function, and inflammatory or stress signaling. We therefore interpret enrichment of neurodegenerative disease‐associated gene sets as evidence that *Plcg2* perturbation affects cellular programs that overlap with those disrupted in neurodegenerative disease, rather than as evidence of AD pathology or a direct disease mechanism. The gene‐level patterns contributing to the mmu05022 enrichment similarly included genes involved in proteostasis, mitochondrial function, and inflammatory/stress signaling (Figure S10), providing biological context for the pathway‐level signal. The broad directional consistency observed across genes and microglial clusters (Figure 5E) further supports coordinated alteration of cellular programs represented within this pathway annotation rather than an effect driven by only a small subset of genes. The present findings consequently provide hypotheses for future studies testing whether these baseline *Plcg2*‐associated programs are maintained, amplified, or altered during aging and neurodegenerative pathology.

Although *Plcg2* expression is predominantly limited to immune cells, our analyses also identified genotype‐associated gene‐expression changes in astrocytes. Mouse‐level astrocyte pseudobulk analyses identified genotype‐associated pathway changes and enrichment among genes ranked by the genotype‐by‐sex interaction statistic (Figure S13), while sex‐stratified mouse‐level and exploratory cell‐level analyses identified additional directional differences between male and female astrocytes (Figure S14 and Figure 7). These results extend the consequences associated with *Plcg2* deficiency beyond microglial expression profiles. Emerging evidence suggests that *Plcg2* may regulate cell types beyond microglia in the brain. Recent work demonstrated that neuronal *Plcg2* downregulation alters dendritic morphology, synaptic function, and neuronal activity independently of direct microglial transduction (Coulon et al. 2026). Other studies have also identified low but functionally relevant expression of *Plcg2* in neurons (Magno et al. 2019; Coulon et al. 2024). *Plcg2* KO brain tissue also exhibits altered expression of myelin‐ and astrocyte‐associated genes in a recent pre‐print (Hopp et al. 2023). Microglia and astrocytes communicate through numerous cytokine, chemokine, growth‐factor, and other signaling pathways both in physiological and pathological conditions (Vainchtein and Molofsky 2020; Garland et al. 2022; Teh et al. 2017), providing one potential explanation for coordinated changes across these populations. Exploratory CellChat analysis identified candidate differences in predicted microglia–astrocyte ligand–receptor communication following *Plcg2* loss (Figure 8 and Figure S16). However, CellChat does not explicitly model inter‐mouse variability in its standard implementation and infers communication from expression patterns rather than directly measuring signaling. We therefore interpret these results as hypothesis‐generating predictions rather than replicate‐level evidence of differential communication between experimental groups. Because *Plcg2* expression was minimal to absent in astrocytes in our dataset (Figure S1B), the astrocytic gene‐expression changes observed here may reflect indirect responses to altered signaling from microglia or other cell populations rather than a cell‐autonomous effect of *Plcg2* loss. The germline model used here cannot distinguish between these possibilities. Future studies using cell type‐specific *Plcg2* perturbation will be necessary to resolve them, while spatial, co‐culture, or other orthogonal approaches will be important for validating predicted intercellular interactions and their functional consequences.

A broader consideration is that the whole‐animal *Plcg2*‐deficient model may influence the brain through developmental and systemic mechanisms in addition to local glial effects. PLCG2 has well‐established roles in peripheral immune cells, including B cells, NK cells, mast cells, neutrophils, macrophages, and platelets, where it regulates receptor‐mediated calcium signaling and diverse immune effector functions (Jackson et al. 2021; Jakus et al. 2009; Wang et al. 2000; Wen et al. 2002). Germline *Plcg2* deficiency also disrupts blood–lymphatic vascular separation during development (Ichise et al. 2009) and is associated with substantial prenatal and preweaning mortality, with lower survival in females than males reported in one *Plcg2* KO cohort (Hopp et al. 2023). Thus, the surviving KO animals analyzed here may represent a developmentally selected population, and some transcriptomic differences attributed to *Plcg2* loss could reflect developmental adaptation or selection rather than solely acute loss of PLCG2 signaling in mature microglia. This potential survival bias, particularly in the context of reported sex differences in KO survival, should be considered when interpreting genotype‐ and sex‐associated transcriptomic changes. Moreover, altered peripheral immune function could indirectly influence the CNS through changes in systemic inflammatory signaling or neuroimmune interactions (Prinz and Priller 2017; Di Pietro et al. 2026). Accordingly, some genotype‐associated brain expression patterns may reflect a combination of CNS‐intrinsic, developmental, and systemic consequences of *Plcg2* deficiency. Future studies using inducible deletion in adult animals will be important for separating these effects.

The number of biological replicates also defines the resolution at which the statistical findings can be interpreted. With 2–3 mice per sex‐by‐genotype group, the study has greater sensitivity to larger genotype effects and coordinated pathway‐level patterns than to moderate individual‐gene genotype‐by‐sex interactions. Accordingly, the current design is likely underpowered to resolve moderate gene‐level interactions, while remaining informative for detecting coordinated effects distributed across biological pathways. We therefore based all primary statistical inference on mouse‐level pseudobulk analyses, including combined‐sex genotype tests, formal genotype‐by‐sex interaction models, and mouse‐level sex‐stratified comparisons. Although no individual microglial genes reached FDR significance for the interaction term, GSEA of genes ranked by the interaction statistic identified coordinated pathway‐level enrichment. We used the cell‐level differential‐expression analyses, cell‐level module‐trait associations, and CellChat analyses as complementary higher‐resolution approaches to identify candidate genes, co‐expression programs, and predicted signaling relationships underlying these mouse‐level patterns. Replication of this study in larger cohorts will be necessary to establish the reproducibility of the sex‐associated patterns, and orthogonal approaches will be required to determine the functional significance of the prioritized candidates.

Whole‐hemisphere sampling was used to maximize cell yield, viability, and glial representation from each animal. Consequently, the findings represent aggregate whole‐hemisphere glial expression patterns and cannot be assigned to individual brain regions. Region‐specific profiling or spatial transcriptomic approaches will be needed to determine whether the observed patterns are broadly distributed or spatially restricted. The age of the mice provides an additional boundary for interpretation. Although microglia are transcriptionally mature in six‐week‐old mice (Attaai et al. 2018; Nikodemova et al. 2015; Hammond et al. 2019), these mice remain young and do not model the aging context in which AD develops. Ongoing developmental processes may therefore contribute to the observed expression patterns, and the cross‐sectional design cannot determine whether these patterns persist, diminish, or change with aging. Within this defined scope, the present dataset provides a baseline, cell‐resolved framework for understanding how partial and complete *Plcg2* loss is associated with glial gene‐expression and pathway patterns and for prioritizing the cellular processes most likely to shape PLCG2 biology across genotype and sex.

## Author Contributions

Luke C. Dabin, Cristian Lasagna‐Reeves, Bruce T. Lamb, and Jungsu Kim conceived the study and designed the experiments. Kelly A. Hartigan, Holly N. Kersey, and Luke C. Dabin performed all experiments and analyzed the scRNA‐seq data. Nur Jury‐Garfe provided the mice used in this study and performed genotyping. Ahmad Daniel Sharify and Byungwook Kim assisted during mouse sacrifice and brain dissection. Kelly A. Hartigan, Luke C. Dabin, Stephanie J. Bissel, and Jungsu Kim interpreted and discussed the results. Kelly A. Hartigan and Jungsu Kim wrote the manuscript. All the authors read and approved the manuscript. Artificial intelligence–based language tools were used to assist with editing for clarity and style. All scientific concepts, data generation, and interpretation were produced by the authors.

## Funding

This work was supported by the National Institute of Health (NIH) (U01AG076804, R01AG077829, R01AG071281, R21AG072738, RF1AG074543), Indiana University (T32AG071444), Indiana Medical Scientist/Engineer Training Program (T32GM148382), National Center for Advancing Translational Sciences (UM1TR004402), Alzheimer's Association Fellowship (AARF‐21‐852175), Strategic Research Initiative Fund, Precision Health Initiative Fund, and P. Michael Conneally Professorship.

## Consent

No human subject materials were used in this study.

## Conflicts of Interest

The authors declare no conflicts of interest.

## Supporting information


**Figure S1:** Expression of *Plcg2* and proportion of microglia by sex and genotype. (A) Summary of single‐cell RNA‐sequencing dataset composition. A total of 62,303 cells were analyzed from 14 mice, including *Plcg2* WT (*n* = 4 mice; 18,594 cells), HET (*n* = 5 mice; 17,813 cells), and KO (*n* = 5 mice; 25,896 cells) animals. Cell numbers by sex and major cell type are indicated. (B) Violin plots showing normalized *Plcg2* expression levels across major brain cell populations. *Plcg2* expression was enriched primarily in microglia and peripheral immune cells, with minimal expression detected in other cell types. (C, D) The mean proportion of each cluster relative to the total population based on genotype, WT (*Plcg2+/+*), HET (*Plcg2*+/−) and KO (*Plcg2*−/−) shown separately for male (C) and female (D) microglia. Points overlaid on bars represent individual biological replicates. Error bars indicate standard error of the mean (SEM). *p adjusted < 0.05.

**Figure S2:** Microglial cluster annotation. (A, B) Ridge plots visualizing UCell scores for homeostatic‐associated (A) and inflammatory‐associated (B) gene module expression across microglial subclusters. Each line represents a distinct microglia subcluster, with color‐coded identifiers. (C, D) Bar plots of enriched biological pathways for marker genes in microglial subclusters 5_M (C) and 8_M (D). The *x*‐axis represents enrichment significance as −log10(Benjamini Hochberg‐adjusted *p*‐value). Bar color indicates gene ratio, defined as the proportion of input genes overlapping with each pathway gene set. Numbers in parentheses indicate the number of overlapping genes contributing to each enriched pathway term.

**Figure S3:** Genotype‐by‐sex interaction analyses for Plcg2 KO and HET versus WT microglia. (A) MSigDB Hallmark GSEA of genes ranked by the KO‐by‐sex interaction Wald statistic in microglia. (B) KEGG GSEA of genes ranked by the HET‐by‐sex interaction Wald statistic. (C) MSigDB Hallmark GSEA of genes ranked by the HET‐by‐sex interaction Wald statistic.

**Figure S4:** Sex‐stratified analysis of Plcg2 genotype effects on the female microglial transcriptome. (A) Volcano plot of differentially expressed genes in female pseudobulk microglia comparing Plcg2 HET versus WT. Average log2 fold change is plotted against −log10(*p*‐value); select top up‐ and downregulated genes are labeled. The horizontal dashed line indicates the nominal *p* = 0.05 display threshold; vertical dashed lines indicate log2 fold change of |0.58|. (B) KEGG pathway GSEA of the female HET versus WT comparison. Dot size indicates gene set size; color indicates *q*‐value; *x*‐axis represents normalized enrichment score (NES). (C) Volcano plot of differentially expressed genes in female pseudobulk microglia comparing Plcg2 KO versus WT, plotted as in (A). (D) KEGG pathway GSEA of the female KO versus WT comparison. (E) MSigDB Hallmark pathway GSEA of the female KO versus WT comparison. For (D, E), dot size indicates gene set size, color indicates *q*‐value, and *x*‐axis represents NES.

**Figure S5:** Cell‐level differential gene expression and pathway enrichment analyses in female *Plcg2* KO versus HET microglia. (A) Scatter plot showing shared differentially expressed genes (DEGs) between female *Plcg2* KO versus WT and HET versus WT microglia. Each point represents a shared DEG, plotted by average log2 fold change (log2FC) in KO/WT (*x*‐axis) and HET/WT (*y*‐axis) comparisons. Genes with concordant, monotonic directional changes are highlighted by quadrant. (B) Volcano plot showing DEGs in female microglia comparing *Plcg2* KO versus HET. Genes meeting significance criteria (Bonferroni adjusted *p*‐value < 0.05, |log2FC| > 0.58) are highlighted, with select genes labeled. The dashed vertical lines indicate fold‐change cutoffs and the horizontal dashed line represents the statistical significance threshold. (C–D) Functional enrichment analyses of differentially expressed genes identified in female *Plcg2* KO versus HET microglia. (C) KEGG pathway enrichment analysis and (D) MSigDB Hallmark pathway enrichment analysis. The *x*‐axis represents enrichment significance as −log10(Benjamini Hochberg‐adjusted *p*‐value). Bar color indicates gene ratio, defined as the proportion of input genes overlapping with each pathway gene set. Numbers in parentheses indicate the number of overlapping genes contributing to each enriched pathway term. The dashed line represents the statistical cutoff for significance (adjusted *p*‐value < 0.05).

**Figure S6:** Sex‐stratified analysis of *Plcg2* genotype effects on the male microglial transcriptome. (A) Volcano plot of differentially expressed genes in male pseudobulk microglia comparing Plcg2 HET versus WT. Average log2 fold change is plotted against −log10(*p*‐value); select top up‐ and downregulated genes are labeled The horizontal dashed line indicates the nominal *p* = 0.05 display threshold; vertical dashed lines indicate log2 fold change of |0.58|. (B) KEGG pathway and (C) MSigDB Hallmark pathway GSEA of the male HET versus WT comparison. Dot size indicates gene set size; color indicates *q*‐value; *x*‐axis represents normalized enrichment score (NES). (D) Volcano plot of differentially expressed genes in male pseudobulk microglia comparing Plcg2 KO versus WT, plotted as in (A). (E) KEGG pathway GSEA of the male KO versus WT comparison. Dot size indicates gene set size, color indicates *q*‐value, and *x*‐axis represents NES.

**Figure S7:** Cell‐level differential gene expression and pathway enrichment analyses in male *Plcg2* KO versus HET microglia. (A) Scatter plot showing shared differentially expressed genes (DEGs) between male *Plcg2* KO versus WT and HET versus WT microglia. Each point represents a shared DEG, plotted by average log2 fold change (log2FC) in KO/WT (*x*‐axis) and HET/WT (*y*‐axis) comparisons. Genes with concordant, monotonic directional changes are highlighted by quadrant. (B) Volcano plot showing DEGs in male microglia comparing *Plcg2* KO versus HET. Genes meeting significance criteria (Bonferroni adjusted *p*‐value < 0.05, |log2FC| > 0.58) are highlighted, with select genes labeled. The dashed vertical lines indicate fold‐change cutoffs and the horizontal dashed line represents the statistical significance threshold. (C–D) Functional enrichment analyses of differentially expressed genes identified in male *Plcg2* KO versus HET microglia. (C) KEGG pathway enrichment analysis and (D) MSigDB Hallmark pathway enrichment analysis. The *x*‐axis represents enrichment significance as −log10(Benjamini Hochberg‐adjusted *p*‐value). Bar color indicates gene ratio, defined as the proportion of input genes overlapping with each pathway gene set. Numbers in parentheses indicate the number of overlapping genes contributing to each enriched pathway term. The dashed line represents the statistical cutoff for significance (adjusted *p*‐value < 0.05).

**Figure S8:** Sex‐stratified MSigDB pathway enrichment across male and female Plcg2 microglia. Heatmap showing significant enrichment for top shared MSigDB pathways among differentially expressed genes (DEGs) in KO and HET microglia compared to sex‐matched WT microglia. Pathways are sorted and colored by Benjamini Hochberg‐adjusted *p*‐value, with darker green indicating greater enrichment significance and white indicating no significant enrichment for that comparison.

**Figure S9:** Intersection and pathway enrichment analysis of cell‐level differentially expressed genes (DEGs) across KO and HET conditions in male and female microglia. (A, C, E) UpSet plots showing intersection sizes among upregulated and downregulated DEGs across KO and HET comparisons relative to sex‐matched WT controls. Shared DEGs exhibiting concordant fold‐change directions are highlighted in the corresponding panels for KO (A) and HET (E) comparisons, whereas DEGs exhibiting opposite fold‐change directions between groups are highlighted in (C). Colored bars denote highlighted DEG intersections of interest. (B, D, F) Bar plots displaying significantly enriched KEGG pathways among DEG sets identified in the corresponding intersection analyses: KO shared DEGs with concordant regulation (B), HET DEGs with opposite fold‐change directions (D), and HET shared DEGs with concordant regulation (F). The *x*‐axis represents enrichment significance as −log10(Benjamini Hochberg‐adjusted *p*‐value). Bar color indicates gene ratio, defined as the proportion of input genes overlapping with each pathway gene set. Numbers in parentheses indicate the number of overlapping genes contributing to each enriched pathway term. The dashed line represents the statistical cutoff for significance (adjusted *p*‐value < 0.05).

**Figure S10:** Sex‐stratified expression changes in proteostasis, mitochondrial dysfunction, and inflammatory signaling genes in *Plcg2* KO microglia. (A, B) Box plots showing normalized mean expression per animal for selected genes associated with proteostasis, mitochondrial dysfunction, and inflammatory/stress signaling pathways in female (A) and male (B) microglia comparing WT and *Plcg2* KO mice. Each point represents an individual biological replicate, colored by sample identity. Boxes indicate median and interquartile range, with whiskers representing the minimum and maximum values across samples.

**Figure S11:** hdWGCNA module identification and characterization in glial cells (A) Dendrogram generated from hdWGCNA analysis delineating four gene co‐expression modules, indicated by colored bar segments. (B) UMAP feature plots showing single‐cell expression scores for each module across cell populations. (C) Dot plot displaying scaled expression of modules across annotated microglial subclusters, with dot size corresponding to the percent of cells expressing module genes. (D, E) Network diagrams showing top hub genes identified in Module 1 (D) and Module 3 (E), alongside bar plots displaying significantly enriched Gene Ontology (GO) biological processes associated with the 50 top hub genes in each module by kME. The *x*‐axis represents enrichment significance as −log10(Benjamini Hochberg‐adjusted *p*‐value). Bar color indicates gene ratio, defined as the proportion of hub genes overlapping with each GO term. Numbers in parentheses indicate the number of overlapping genes contributing to each enriched GO process. The dashed line represents the statistical cutoff for significance (adjusted *p*‐value < 0.05).

**Figure S12:** Cell type‐specific hdWGCNA module–trait correlations. Heatmap showing correlations between hdWGCNA co‐expression module eigengenes (Modules 1–4, color‐coded at bottom) and experimental traits (sex, Plcg2 genotype [KO, HET], and specific sex‐genotype indicator variables [male‐KO and male‐HET]) across cell types (all cells, microglia, ependymal, endothelial, astrocytes, peripheral immune, red blood cells [RBCs], and neurons). Color indicates the strength and direction of the Pearson correlation between module eigengene expression and each trait, with red indicating positive correlation and blue indicating negative correlation (scale, −0.2 to 0.2). Asterisks denote Benjamini‐Hochberg‐adjusted *p*‐values (·*p* < 0.1, *p < 0.05, **p < 0.01, ***p < 0.001).

**Figure S13:** Plcg2 genotype and sex‐by‐genotype interaction analyses in astrocytes. (A, C) Volcano plots of pseudobulk differential‐expression results in astrocytes comparing Plcg2 HET versus WT (A) and KO versus WT (C). Genes meeting the nominal display threshold (*p*‐value < 0.05, |log2FC| > 0.58) are highlighted, with select genes labeled; genes additionally reaching a Benjamini‐Hochberg‐adjusted *p*‐value < 0.05 are shown in darker color. Red indicates genes upregulated in HET/KO; blue indicates genes upregulated in WT. (B, D) KEGG pathway GSEA of genes ranked by the signed Wald statistic from the HET versus WT (B) and KO versus WT (D) comparisons. (E, F) KEGG pathway GSEA of genes ranked by the HET‐by‐sex interaction Wald statistic (E) and KO‐by‐sex interaction Wald statistic (F) in astrocytes. For (B, D, E, F), the *x*‐axis represents the normalized enrichment score (NES); dot size indicates gene set size, and color indicates *q*‐value.

**Figure S14:** Sex‐stratified analysis of *Plcg2* genotype effects on the astrocyte transcriptome. (A, C) Volcano plots showing differentially expressed genes (DEGs) in female pseudobulk astrocytes comparing *Plcg2* HET versus WT (A) and KO versus WT (C). Genes meeting the nominal display threshold (*p*‐value < 0.05, |log2FC| > 0.58) are highlighted, with select genes labeled; genes additionally reaching a Benjamini‐Hochberg‐adjusted *p*‐value < 0.05 are shown in darker color. Red indicates genes upregulated in HET/KO; blue indicates genes upregulated in WT. (B, D) KEGG pathway GSEA of genes ranked by the corresponding signed Wald statistic from the female HET versus WT (B) and KO versus WT (D) comparisons. (E, G) Volcano plots showing DEGs in male pseudobulk astrocytes comparing *Plcg2* HET versus WT (E) and KO versus WT (G), plotted as in (A, C). (F, H) KEGG pathway GSEA of genes ranked by the corresponding signed Wald statistic from the male HET versus WT (F) and KO versus WT (H) comparisons. For (B, D, F, H), the *x*‐axis represents the normalized enrichment score (NES); dot size indicates gene set size, and color indicates *q*‐value.

**Figure S15:** Sex‐stratified astrocyte gene‐expression patterns in *Plcg2* HET mice. (A, B) Volcano plots showing differentially expressed genes (DEGs) in female (A) and male (B) astrocytes comparing *Plcg2* HET versus WT mice. Genes meeting significance criteria (Bonferroni adjusted *p*‐value < 0.05, |log2FC| > 0.58) are highlighted, with select genes labeled. The dashed vertical lines indicate fold‐change cutoffs and the horizontal dashed line represents the statistical significance threshold. (C) Heatmap showing significant enrichment for shared KEGG pathways among astrocyte DEGs identified in female KO, female HET, and male KO comparisons relative to sex‐matched WT controls. Pathways are sorted and colored by Benjamini Hochberg‐adjusted *p*‐value, with darker green indicating greater enrichment significance and white indicating no significant enrichment for that comparison.

**Figure S16:** Sex‐stratified differences in predicted microglia–astrocyte communication following *Plcg2* loss. (A, B) Chord diagrams showing predicted ligand–receptor interactions between microglia and astrocytes in KO versus WT samples. (A) Predicted interactions with higher communication probability in female KO versus WT samples and (B) predicted interactions with lower communication probability in male KO versus WT samples. Colored sectors represent ligand and receptor genes associated with each cell type, and connecting ribbons indicate predicted ligand–receptor interactions. Ribbon width reflects inferred interaction strength based on communication probability. Red denotes microglia and purple denotes astrocytes.

**Table S1:** Per‐animal, per‐sex, per‐genotype cell counts stratified by cluster.

**Table S2:** Marker gene list for the clusters identified in scRNA‐seq analysis, related to Figure 1A.

**Table S3:** Proportional analysis of cell types via scRNAseq, related to Figure 1C.

**Table S4:** Proportional analysis of microglia clusters, split by sex, related to Figure S1C,D.

**Table S5:** Expression of Plcg2 and Alzheimer's disease‐associated genes across identified microglial clusters, Related to Figure 1E.

**Table S6:** Marker gene list for 5_M and 8_M as compared to other microglial clusters, related to Figure S2C,D.

**Table S7:** Pathway analysis of 5_M and 8_M. Related to Figure S2C,D.

**Table S8:** Psuedobulked DEGs between genotypes, controlled for sex, related to Figure 2A–F.

**Table S9:** Gene set enrichment analysis of pseudobulked DEGs in microglia—KO/WT, related to Figure 2B,C.

**Table S10:** Gene set enrichment analysis of pseudobulked DEGs in microglia—HET/WT, related to Figure 2E,F.

**Table S11:** Psuedobulked sex‐by‐genotype interaction effect, related to Figure S3.

**Table S12:** Gene set enrichment analysis of microglial interaction effect—sex:genotype (KO vs. WT), Related to Figure 2H and Figure S3A.

**Table S13:** Gene set enrichment analysis of microglial interaction effect—sex:genotype (HET vs. WT), related to Figure S3B,C.

**Table S14:** Psuedobulked DEGs between genotypes, female microglia, related to Figure S4A,C.

**Table S15:** Gene set enrichment analysis of pseudobulked DEGs in female microglia—HET/WT, related to Figure S4B.

**Table S16:** Gene set enrichment analysis of pseudobulked DEGs in female microglia—KO/WT, related to Figure S4D,E.

**Table S17:** DEGs between genotypes for all Female Microglia, related to Figure 3A,B and Figure S5A,B.

**Table S18:** Female microglial DEGs, shared and unique between KO and HET comparisons, related to Figure 3D.

**Table S19:** Female monotonic genes (|KO/WT| > |HET/WT|), related to Figure S5A.

**Table S20:** Pathway analysis of DEGs in female microglia—HET/WT, related to Figure 3E,F.

**Table S21:** Pathway analysis of DEGs in female microglia—KO/WT, related to Figure 3G,H.

**Table S22:** Pathway analysis of DEGs in female microglia—KO/HET, related to Figure S5C,D.

**Table S23:** Psuedobulked DEGs between genotypes, male microglia, related to Figure S6A,D.

**Table S24:** Gene set enrichment analysis of pseudobulked DEGs in male microglia—HET/WT, related to Figure S6B,C.

**Table S25:** Gene set enrichment analysis of pseudobulked DEGs in male microglia—KO/WT, related to Figure S6E.

**Table S26:** DEGs between genotypes for all Male Microglia, related to Figure 4A,B and Figure S7B.

**Table S27:** Male Microglial DEGs, shared and unique between KO and HET comparisons, related to Figure 4D.

**Table S28:** Male Monotonic Genes (|KO/WT| > |HET/WT|), related to Figure S7A.

**Table S29:** Pathway analysis of DEGs in male microglia—HET/WT, related to Figure 4E,F.

**Table S30:** Pathway analysis of DEGs in male microglia—KO/WT, related to Figure 4G,H.

**Table S31:** Pathway analysis of DEGs in male microglia—KO/HET, related to Figure S7C,D.

**Table S32:** Intersections between male and female microglial DEGs, related to Figure 5B,C, Figure S9A,C,E.

**Table S33:** KEGG Pathway analysis of DEGs in microglia, related to Figure 5D; Figure S9B,D,F.

**Table S34:** Log2FC values of KEGG Neurodegeneration gene set, split by sex and cluster, related to Figure 5E

**Table S35:** hdWGCNA module assignments, related to Figure 6 and Figure S11.

**Table S36:** hdWGCNA differential module expression between KO and WT, both sexes, related to Figure 6B.

**Table S37:** Pathway analysis of top hub genes per module, related to Figure 6D,F and Figure S11D,E.

**Table S38:** Psuedobulked DEGs between genotypes, controlled for sex, related to Figure S13A–D.

**Table S39:** Gene set enrichment analysis of pseudobulked DEGs in astrocytes, controlled for sex, related to Figure S13B,D.

**Table S40:** Psuedobulked sex‐by‐genotype interaction effect, related to Figure S13E,F.

**Table S41:** Gene Set Enrichment Analysis of Astrocyte Interaction Effect‐ sex:genotype, related to Figure S13E,F.

**Table S42:** Psuedobulked DEGs between genotypes, stratified sex comparisons for astrocytes, related to Figure S14A,C,E,G.

**Table S43:** Gene Set Enrichment Analysis of sex‐stratified Astrocyte comparisons, related to Figure S14B,D,F,H.

**Table S44:** DEGs between genotypes for all Astrocytes, related to Figure 7A,B and Figure S15A,B.

**Table S45:** Pathway analysis of DEGs in astrocytes—KO/WT, related to Figure 7C,D and Figure S15C.

**Table S46:** Pathway analysis of DEGs in astrocytes—HET/WT, related to Figure S15C.

**Table S47:** Intersections between male and female astrocyte DEGs, related to Figure 7E,F.

**Table S48:** KEGG Pathway analysis of sex‐discordant DEGs in astrocytes, related to Figure 7F.

**Table S49:** The differential receptor‐ligand interactions between the clusters identified via CellChat analysis, related to Figure 8A–H and Figure S16A,B.

## Data Availability

Raw data have been deposited in the NIH GEO database, scRNA‐seq data (GSE313890). The code used for the main analysis of this paper will be deposited at GitHub at the time of publication (https://github.com/jungsukimlab). Any additional information required to reanalyze the data reported in this study is available from the lead contact upon request.
